# Protective factors against acute kidney injury in children exposed to diethylene glycol-contaminated medicines: A case-case-control analysis from The Gambia outbreak

**DOI:** 10.1371/journal.pgph.0005512

**Published:** 2026-05-15

**Authors:** Amadou Barrow, Mustapha Bittaye, Sheikh Omar Bittaye, Sunday Musa Oguche

**Affiliations:** 1 Department of Epidemiology, College of Public Health & Health Professions, College of Medicine, University of Florida, Gainesville, Florida, United States of America; 2 Department of Public & Environmental Health, School of Medicine & Allied Health Sciences, The University of the Gambia, Faraba Campus, Faraba, West Coast Region, The Gambia; 3 Edward Francis Small Teaching Hospital, Independence Drives, Banjul, The Gambia; 4 School of Medicine & Allied Health Sciences, The University of the Gambia, Independence Drives, Banjul, The Gambia; Directorate of Factories, INDIA

## Abstract

During The Gambia’s 2022 diethylene glycol (DEG) outbreak, 82 children developed acute kidney injury (AKI) with 80% mortality. However, approximately 30% of children with documented DEG exposure did not develop AKI. Understanding protective factors could enable risk stratification and inform prophylactic interventions in future outbreaks. We conducted a case-case-control study among 321 children aged ≤8 years from six Gambian health regions. We compared exposed children who developed AKI (exposed-susceptible, n = 37) with exposed children who remained healthy (exposed-resistant, n = 16) and unexposed healthy children (n = 242). Multivariable logistic regression identified factors associated with AKI resistance among the 53 exposed children, adjusting for age, sex, and socioeconomic indicators. Sensitivity analyses included propensity score matching and stratification. Three factors were independently associated with reduced AKI risk: older age (adjusted OR=0.58 per year, 95% CI [0.36-0.92], p = .021), multivitamin supplementation (aOR=0.24, 95% CI [0.06-0.85], p = .028), and exposure to a single contaminated medicine (aOR=4.21 for ≥2 vs 1 medicine, 95% CI [1.12-16.85], p = .034). A dose-response relationship was observed, with AKI odds increasing 4-fold per additional contaminated medicine (p-trend = .018). Promethazine oral solution showed strongest toxicity (aOR=4.15, 95% CI [1.15-15.82]), consistent with highest DEG concentration (19.4 mg/mL). Multivitamin effects were strongest in children <18 months (aOR=0.15, 95% CI [0.02-0.81]). Findings remained robust across propensity score-matched analyses. Study limitations include small sample size of resistant children (n = 16), potential recall bias, wide confidence intervals, and possible residual confounding of multivitamin associations by socioeconomic and health literacy factors. Substantial heterogeneity exists in pediatric DEG susceptibility. Younger age, polypharmacy with multiple contaminated products, and absence of multivitamin supplementation were independently associated with increased AKI risk. These findings suggest potential targets for risk stratification and prophylactic strategies, though observed multivitamin associations require prospective validation with attention to confounding before clinical implementation.

## Introduction

Diethylene glycol (DEG) contamination of pharmaceuticals is a catastrophic yet preventable global poisoning event. Since the 1937 U.S. outbreak, where 107 people, mostly children, died from DEG-tainted sulfanilamide elixir [1], at least 15 major outbreaks have occurred on six continents, killing thousands [2–4]. DEG, used in antifreeze and brake fluid, is sometimes substituted for glycerin or propylene glycol in medicine manufacturing due to cost [5]. Ingested DEG is metabolized to diglycolic acid, a nephrotoxic metabolite causing acute tubular necrosis via mitochondrial dysfunction and oxidative stress [6–8]. Clinical presentation includes gastrointestinal symptoms, oliguria, anuria, metabolic acidosis, and acute kidney injury (AKI), with fatality rates often exceeding 70% in pediatric cases [9–12].

Children are disproportionately affected by DEG contamination for several reasons. Liquid formulations like cough syrups and fever reducers, often contaminated, are mainly given to children [13,14]. Children may receive higher doses per kilogram than adults. Their immature renal function and evolving metabolism increase vulnerability to nephrotoxic injury [15,16]. Poisoning symptoms in children, often mistaken for illness, lead to continued exposure and irreversible renal failure before detection [17]. Despite decades of warnings from health authorities [14,18], DEG contamination still causes pediatric deaths, with outbreaks in India (2019–2022) [19], Indonesia, The Gambia, Uzbekistan, Cambodia (2022–2023) [19,20], and Cameroon (2023) [20]. These tragedies highlight systemic failures in pharmaceutical quality assurance, especially in LMICs, where regulatory infrastructure is often inadequate [21,22].

Between June and September 2022, The Gambia faced a severe DEG contamination outbreak affecting children. On June 21, 2022, Maiden Pharmaceuticals, an Indian company, imported four pediatric syrups into The Gambia: Promethazine oral solution, Kofexmalin baby cough syrup, Makoff baby cough syrup, and Magrip N cold syrup. These were distributed nationwide and widely bought for children with respiratory symptoms. In late June, Edward Francis Small Teaching Hospital (EFSTH) noticed a surge in pediatric AKI cases with fever, vomiting, oliguria progressing to anuria, and refractory metabolic acidosis [9,11]. By October 5, 2022, 82 cases were identified with 66 deaths (80.5% case fatality rate), affecting children aged 5 months to 7 years (median age: 19 months) [9]. WHO laboratory analysis found unacceptable DEG levels (range: 7.1–24.8 mg/mL) and ethylene glycol in all four products, while other pediatric medicines showed no contamination [9,23]. The Gambian government recalled the products, banned imports from the manufacturer, and enhanced medicine surveillance. A Ministry of Health assessment concluded that 56 AKI cases and 22 deaths were directly due to DEG-contaminated medicines, with additional likely related cases lacking definitive exposure documentation [23].

While the causal link between the contaminated medicines and the AKI outbreak was established through epidemiological investigation, laboratory testing, and temporal association [9,23], a key question remained: Why did some children consuming the contaminated medicines develop fatal AKI while others were asymptomatic? Among children with documented exposure, about 30% did not develop AKI despite confirmed consumption. Understanding the determinants of this differential susceptibility has significant clinical and public health implications. It could enable risk stratification to identify exposed children at highest risk for intensive surveillance and early intervention. It may also reveal modifiable protective factors for prophylactic interventions in future outbreaks. Additionally, it could provide insights into the biological mechanisms underlying DEG toxicity and individual vulnerability, informing therapeutic strategies. Finally, causal inference would be strengthened by examining dose-response relationships and biological gradients, key criteria in Bradford Hill’s framework for establishing causality [24].

To address this critical knowledge gap, we conducted a case-case-control study comparing three groups of children: those who developed AKI after exposure to contaminated medicines (exposed-susceptible), those who remained healthy despite exposure (exposed-resistant), and unexposed healthy controls (unexposed-healthy). Our primary objective was to identify demographic, clinical, nutritional, and exposure-related factors associated with protection against AKI among exposed children. Secondary objectives included examining dose-response relationships between contaminated medicines and AKI risk, assessing medicine-specific toxicity profiles, and investigating potential effect modification by age and concomitant medication use. We hypothesized that resistance to DEG toxicity would be linked to older age (indicating greater renal maturity and enzyme system development), better nutritional status, lower cumulative dose exposure (fewer medicines, shorter duration), and potentially protective concomitant medications, like multivitamins or traditional medicines. This study is the first to investigate protective factors in pediatric DEG poisoning, providing actionable evidence for clinical management as these outbreaks tragically continue.

## Methods

### Study design and data source

This study employed a case-case-control design to investigate the protective factors against AKI among children exposed to DEG-contaminated medicines during The Gambia’s 2022 outbreak. We conducted a secondary analysis of data originally collected for a case-cohort study examining the causes and risk factors of the AKI outbreak [10]. The case-cohort study, conducted by the Gambian Ministry of Health in collaboration with the WHO Regional Office for Africa, enrolled participants between December 15 and 22, 2022, approximately 3–6 months after the outbreak period (June-September 2022).

The case-case-control design was chosen for this analysis due to its superior statistical efficiency in identifying effect modifiers and protective factors compared to traditional designs [25]. This approach is valuable when DEG-contaminated medicines are already established as causal, and the focus shifts to response heterogeneity among exposed individuals [26,27]. By comparing exposed children who developed AKI to those who remained healthy, we examined factors modifying susceptibility to DEG toxicity while controlling for exposure. Including unexposed healthy children provided context and enabled sensitivity analyses using multinomial regression.

### Study setting

The study was conducted in six of The Gambia’s seven health regions: Western Region 1 (WR1), Western Region 2 (WR2), Central River Region (CRR), North Bank West Region (NBWR), Lower River Region (LRR), and Upper River Region (URR). The North Bank East Region (NBER) was excluded because no AKI cases were reported from that region during the outbreak period. The Gambia is a small West African nation with a population of approximately 2.4 million, of which one-third are children < 8 years of age [28]. The country has a single tertiary referral hospital, EFSTH in Banjul, where the majority of AKI cases were diagnosed and managed. The outbreak occurred during The Gambia’s rainy season (June-September), a period typically characterized by increased respiratory infections and healthcare utilization for common childhood illnesses.

### Study population and participant selection

The target population comprised all children aged ≤ 8 years residing in The Gambia during the outbreak period (June-September 2022). The source population consisted of children from the six study regions, from which cases and controls were identified. The original case-cohort study enrolled a total of 321 participants: 63 AKI cases and 258 controls without AKI [10]. For the present secondary analysis, we reclassified these 321 participants into three distinct analytical groups based on disease status (AKI present/absent) and documented exposure to DEG-contaminated medicines (exposed/unexposed):

*Group 1 (exposed-susceptible):* Children who had documented exposure to at least one of the four implicated contaminated medicines (Promethazine oral solution BP, Kofexmalin baby cough syrup, Makoff baby cough syrup, or Magrip N cold syrup) and developed AKI during the outbreak period. This group represents cases among the exposed population.

*Group 2 (exposed-resistant):* Children with documented exposure to at least one of the four implicated contaminated medicines but did not develop AKI. This group represented the “resistant” phenotype and was the key group of interest, as understanding its characteristics could reveal protective factors.

*Group 3 (unexposed-healthy):* Children with no documented exposure to any of the four implicated contaminated medicines and did not develop AKI. This group served as the reference population for contextual analyses.

**Fig 1:**
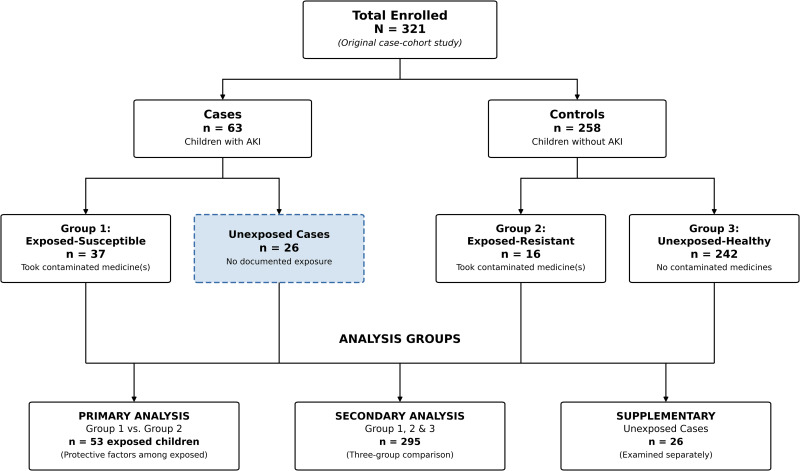
Study flow diagram. *Legend:* AKI = acute kidney injury. Contaminated medicines = Promethazine oral solution, Kofexmalin, Makoff, or Magrip N. Exposed-Susceptible (Group 1) = children who took contaminated medicine(s) and developed AKI. Exposed-Resistant (Group 2) = children who took contaminated medicine(s) but did not develop AKI. Unexposed-Healthy (Group 3) = children who did not take contaminated medicines and had no AKI. Dashed boxes indicate exclusion from primary analysis.

An additional 26 children with AKI but without documented exposure to the implicated medicines were identified but excluded from the primary analysis comparing groups 1 and 2. These “unexplained cases” were examined separately in supplementary analyses to assess potential exposure misclassification or alternative causes of AKI. The study flow diagram (Fig 1) illustrates participant allocation across groups. All 53 exposed children were included in the primary analysis to examine the factors associated with resistance to AKI among those exposed to contaminated medicines.

### Case and control definitions

#### Acute Kidney Injury case definition.

The case definition for AKI was adapted from the previous AKI studies [9,10,29], which was itself based on WHO guidelines for outbreak investigation in resource-limited settings [30] and modified KDIGO criteria for pediatric AKI [31]. Given the outbreak emergency context and limited laboratory capacity in The Gambia (only one reference laboratory nationally), a tiered case definition was employed to balance sensitivity (capturing all true cases) with specificity (minimizing false positives):

*Suspected AKI case:* A child aged ≤8 years presenting with any of the following symptom combinations during the outbreak period (June-September 2022): (1) fever, vomiting, diarrhea, or cough with history of syrup consumption; (2) fever, vomiting, diarrhea, or cough with reduced urine output within 24 hours; or (3) reduced urine output within 24 hours regardless of other symptoms.

*Probable AKI case:* Any suspected case in which the patient died before laboratory confirmation could be obtained. This category was necessary because many children presented in extremis or died within hours of presentation, precluding serum creatinine measurement. The high case-fatality rate (80%) and rapid disease progression meant that excluding these cases would substantially underestimate outbreak magnitude and introduce survivor bias.

*Confirmed AKI case:* Any suspected case with either (1) acute onset of oliguria (urine output <0.5 mL/kg/hr for >6 hours) or anuria (urine output <100 mL/24 hours) of unknown etiology lasting >24 hours, consistent with modified pRIFLE criteria [32]; or (2) elevated serum creatinine levels (measured creatinine >1.5-2 times the age-specific reference range) with no alternative explanation, consistent with KDIGO Stage 2–3 AKI [31].

For this secondary analysis, we operationally defined “AKI” as any child meeting the confirmed or suspected case definition who was enrolled in the original study as a case. The primary analysis included both confirmed cases (n = 58, 92.1% of all cases) and suspected cases (n = 5, 7.9% of all cases) to maximize statistical power, with a sensitivity analysis limited to confirmed cases only to assess robustness of findings.

#### Exposure definition.

Exposure to DEG-contaminated medicines was identified using a composite variable (“exposure_adulterated_med”), classifying children as exposed if caregivers reported consumption of any of the four implicated medicines during the outbreak [10]. Exposure was determined through structured interviews with primary caregivers, mainly mothers, recalling medicine names, brands, and formulations given to children from June to September 2022. Interviewers used prompts like the Tobaski festival (July 2022) for recall and showed medicine bottle pictures when available. We created several variables for dose-response and medicine-specific analyses. Number of adulterated medicines: A count variable (range: 0–4) summing exposure to Promethazine, Kofexmalin, Makoff, and Magrip. This was categorized as single medicine (1) versus polypharmacy (≥2) for primary analyses. Specific medicine exposures: Four binary variables for each contaminated medicine (Promethazine, Kofexmalin, Makoff, Magrip). Total medication burden: A continuous variable summing all unique medications (contaminated and non-contaminated) reported during the outbreak, serving as a proxy for cumulative drug exposure.

#### Data collection procedures.

The original case-cohort study’s data collection procedures are detailed elsewhere [10]. Research assistants fluent in local languages conducted face-to-face interviews with primary caregivers using a structured questionnaire via the KoboCollect platform. The questionnaire was pre-tested and revised. Interviews were conducted in participants’ homes, with informed consent from caregivers. Cases were identified through hospital registry surveillance in six regions, focusing on EFSTH. All children diagnosed with AKI between June and September 2022 were included. Controls were selected using probability proportional to size sampling from the same villages, with random household selection. The strategy aimed for three controls per case for efficiency. The complete dataset from the original study was used, including all 321 participants, reclassified into three groups based on exposure and outcome.

### Variables and measurements

#### Primary outcome.

The primary outcome of this analysis was the occurrence of AKI (binary yes/no) among children with documented exposure to DEG-contaminated medicines. Among the 53 exposed children, the outcome differentiated Group 1 (exposed-susceptible, AKI present, n = 37) from Group 2 (exposed-resistant, AKI absent, n = 16).

#### Primary exposures and protective factors.

We examined a comprehensive set of potential protective factors and risk modifiers:

*Demographic variables:*

- Age in months (continuous)
- Age categories (<12, 12–23, 24–35, ≥ 36 months)
- Sex (male/female)
- Geographic region (WR1, WR2, URR, other regions)
- Urban versus rural residence

*Socioeconomic indicators:*

- Composite income score (0–6 points) constructed from:
- Primary water source (piped, tube well/borehole, or surface water)
- Electricity availability (yes/no)
- Refrigerator ownership (yes/no)
- Television ownership (yes/no)
- Income categories: low (<3), middle (3–4), high (>4)
- Maternal education (none/Quranic only, primary/secondary, tertiary)
- Paternal education (when available)
- Household head’s occupation

*Anthropometric and nutritional indicators:*

- Weight (kg)
- Height/length (cm)
- Mid-upper arm circumference (MUAC, cm)
- Weight-for-age, height-for-age, and weight-for-height z-scores (WHO growth standards)
- Binary malnutrition indicator (any of: weight-for-age z-score < -2, height-for-age z-score < -2, or MUAC <12.5 cm)

*Medical history:*

- Prematurity (gestational age < 37 weeks per maternal report)
- Previous AKI episodes
- Prior hospitalizations
- Prior surgical procedures

*Concomitant medication exposures:*

- Any antibiotic use
- Specific antibiotics: amoxicillin, ampicillin, gentamicin, ceftriaxone, ciprofloxacin, cotrimoxazole
- Any anti-inflammatory use
- Specific agents: acetaminophen/paracetamol, ibuprofen, diclofenac, aspirin
- Multivitamin supplementation
- Traditional oral medicines
- Other cough syrups (not among the four implicated products)
- Composite “concomitant drugs” variable (antibiotics, anti-inflammatories, or antimalarials)

*Clinical presentation:*

Binary variables (yes/no) for 15 symptoms: fever, cough, vomiting, loss of appetite, runny nose, anuria, abdominal pain, chest pain, rash, shortness of breath, malaria diagnosis, wheezing, bloody diarrhea, palpitations, jaundice/discoloration

*Environmental exposures:*

- Residential flooding
- Pesticide exposure
- Herbicide exposure
- Industrial chemical exposure

#### Derived variables.

Several variables were derived specifically for this study. *Age groups:* We created multiple age categorizations to examine non-linear age effects: very young (<18 months), young (18–35 months), and older (≥36 months); alternatively, < 24 months versus ≥24 months for stratified analyses. *Polypharmacy:* A dichotomous variable indicating exposure to ≥2 contaminated medicines (yes/no), with single medicine exposure as the reference. *Medication burden categories:* Total unique medications were categorized as low (0–2 medications), moderate (3–5 medications), or high (≥6 medications). *Caregiver type*: A binary variable indicating whether the primary caregiver was the biological mother (yes/no), as non-maternal caregivers might provide less accurate medication histories.

### Sample size and power

The original case-cohort study was powered to detect the overall association between contaminated medicine exposure and AKI, with a planned sample size of 625 children (500 in the main cohort and 125 buffer for non-response) [10]. For the present secondary analysis, our effective sample size for the primary comparison (Groups 1 and 2) was 53 exposed children (37 susceptible and 16 resistant). Post-hoc power calculations indicated 80% power to detect odds ratios ≥3.0 for exposures with 50% prevalence in the susceptible group and 20% prevalence in the resistant group, using a two-tailed α = 0.05. We therefore focused our interpretation on large, clinically meaningful effect sizes and acknowledge limited power for detecting moderate effects (OR 1.5-2.5).

### Statistical analysis

Descriptive Analysis: Descriptive statistics for all variables in three groups (exposed susceptible, exposed resistant, unexposed healthy) included medians and IQR for skewed distributions, and means with SD for literature comparison. Categorical variables were summarized with frequency and percentage. Differences were assessed using Kruskal-Wallis tests for continuous variables and chi-square or Fisher’s exact tests (when expected cell counts <5) for categorical variables. The primary comparison was Group 1 versus Group 2, using Mann-Whitney U tests (continuous variables) or Fisher’s exact tests (categorical variables) to identify protective factors. Univariable analysis: For each potential protective factor, crude odds ratios (cORs) with 95% confidence intervals (CIs) were calculated comparing exposed-susceptible (Group 1) and exposed-resistant (Group 2). Odds ratios >1.0 indicated increased AKI susceptibility, whereas ORs < 1.0 indicated potential protective factors. Unconditional logistic regression was used for common exposures and Fisher’s exact test for rare exposures (cell counts <5). Variables with p-values ≤0.20 in the univariable analysis were candidates for multivariable modeling, consistent with established variable selection approaches for epidemiological analyses [33,34]. The complete univariable results for all variables examined are reported in Table I in S1 Appendix.

Multivariable analysis: Logistic regression models with AKI (yes/no) as the outcome were used among 53 exposed children. Variables with p ≤ 0.20 in univariable screening were included. Multicollinearity was checked using variance inflation factors (VIF), with VIF > 5 indicating issues [35]. For collinear variables (e.g., contaminated medicines, medicine types, total medication burden), separate models were created. Backward stepwise elimination retained variables with p ≤ 0.05. Age and sex were included in all models as confounders [36]. Interactions between key variables (age × number of medicines, age × multivitamin use, multivitamin × number of medicines) were tested using likelihood ratio tests with p ≤ 0.10; full interaction model results are presented in Table G in S1 Appendix. Model fit was assessed using the Hosmer-Lemeshow test (adequate fit if p > 0.05) [37]and discriminatory ability using AUC (AUC > 0.80 indicates excellent discrimination) [38]. Influential observations were checked using standardized residuals and leverage statistics, with none meeting exclusion criteria (standardized residual >3 or leverage >2(k + 1)/n, where k = number of predictors). Nested models were compared using AIC and BIC, with lower values indicating better fit [39]. Non-nested models were compared using AUC, and McFadden’s pseudo-R² was calculated to quantify variance explained. Primary models: Model 1 included age (per 12-month increase), sex, number of adulterated medicines (≥2 vs. 1), and multivitamin use. It examined age, dose-response, and multivitamins’ protective effects. Model 2 replaced the number of medicines with specific type (promethazine yes/no) to isolate the most toxic drug’s effect, retaining age, sex, and multivitamin use. Model 3 assessed pharmaceutical exposure by including total unique medications with age, sex, and multivitamin use. Model 4 added the age × number of medicines interaction term to see if dose-response varied by developmental stage. Results are presented as adjusted odds ratios (aOR) with 95% CIs and p-values, noting statistical (p < 0.05) and clinical/epidemiological significance, as wide CIs in a small sample might include important effects despite p-values >0.05. All analyses used R version 4.3.2.

To strengthen causal inference, we tested dose-response relationships using two approaches. First, we modeled contaminated medicines as an ordinal variable (1, 2, and ≥3 medicines) and tested linear trends using the score test [40]. Second, we calculated adjusted odds ratios for each exposure level (2 medicines, ≥ 3 medicines) compared to single medicine exposure, adjusting for age and multivitamin use. Results were presented graphically using forest plots, and we calculated the p-value for trend. Given the strong age effect observed and known developmental differences in renal function and drug metabolism [41], we conducted age-stratified analyses. Children were categorized as <18 months, 18–35 months, and ≥36 months based on developmental milestones. Within each stratum, we estimated adjusted odds ratios for key protective factors (multivitamin use and medicine count) using logistic regression, controlling for sex and other covariates where sample size permitted. We present stratum-specific effect estimates with 95% confidence intervals and note where confidence intervals overlap across strata.

### Sensitivity analyses

We conducted six sensitivity analyses (Tables C–E and H in S1 Appendix) to assess robustness. Sensitivity analysis 1: Propensity Score Matching. We estimated propensity scores for Group 2 (exposed-resistant) vs. Group 1 (exposed-susceptible) [42]. Scores were estimated using logistic regression, with Group 2 exposure as the outcome and baseline covariates (age, sex, region, urban residence, income) as predictors. We performed 1:1 nearest-neighbor matching without replacement using a caliper width of 0.2 standard deviations of the logit of the propensity score [43]. Covariate balance post-matching was assessed using standardized mean differences (SMD), with SMD < 0.10 indicating good balance [44]. Treatment effects in the matched sample were estimated using conditional logistic regression. Sensitivity analysis 2: Multiple imputation for missing data. Anthropometric data (weight, height, MUAC) were missing for 68% of participants. We performed multiple imputation using chained equations (MICE) [45] with 20 imputed datasets. The imputation model included analysis variables plus auxiliary variables correlated with missingness (region, income, maternal education, prematurity, flooding exposure). Imputed values were generated using predictive mean matching for continuous variables and logistic regression for binary variables. We combined results across imputations using Rubin’s rules [46] and compared them with complete case analyses.

Sensitivity analysis 3 tested five exposure definitions: (1) any use of four medicines; (2) Promethazine only; (3) any cough syrup including “other” brands; (4) ≥2 medicines from the implicated manufacturer; (5) caregiver report of “took medicine that made child sick.” Findings suggest robustness to misclassification. Sensitivity analysis 4: Confirmed cases only, restricting to confirmed AKI cases (n = 58, excluding 5 suspected) to assess suspected cases’ influence. Sensitivity analysis 5: Multinomial analysis using logistic regression with three outcome categories (Group 3 as reference, Groups 2 and 1 as comparisons) to assess how Exposed-Resistant children compared to unexposed controls and Exposed-Susceptible children, contextualizing protective factors. Sensitivity analysis 6: Geographic clustering examined resistant children’s geographic clustering using spatial analysis techniques. Moran’s I statistic assessed spatial autocorrelation [47] with supplementary analyses using village-level random effects for potential within-village correlations.

### Supplementary analyses

Unexplained cases analysis: We compared the 26 AKI cases without documented exposure to the four drugs with both exposed cases and unexposed controls to assess alternative exposures or misclassification patterns. Medicine-specific toxicity ranking: We constructed a multivariable model including all four medicines as simultaneous predictors to compare their toxicities, treating promethazine (highest crude OR) as the reference. Household-level analysis: For households with multiple enrolled children, we examined concordance in outcomes to assess familial/genetic clustering. See Tables B, F, and H in S1 Appendix for full results.

### Missing data

We assessed the patterns of missingness using Little’s test for missing completely at random (MCAR) [48]. For variables with <20% missing data, we used complete case analysis for primary models. For variables with substantial missingness (>20%), particularly anthropometric measures, we conducted multiple imputations as described above and reported both complete-case and imputed results. For categorical variables with missing categories, we created explicit “missing” indicator categories and examined their distributions but excluded them from the final models if they represented <5% of the sample.

### Ethical considerations

This secondary analysis was conducted under the ethical approvals from the WHO African Regional Office Ethics Review Committee (Protocol ID: AFR/ERC/2022/12.4) and the University of The Gambia Ethics Committee (UTGEC 01.12.22). The original study obtained informed consent from caregivers. No additional participant contacts or data collection was performed for this analysis. Data were de-identified before analysis. This study adhered to the Declaration of Helsinki [49]and followed STROBE guidelines for case-control studies [50]. We report all analyses conducted and acknowledge limitations affecting interpretation.

## Results

### Participant characteristics

A total of 321 children were enrolled in this study, comprising 63 cases with AKI and 258 controls without AKI. Among these participants, 53 children (16.5%) had documented exposure to diethylene glycol (DEG)-contaminated medicines. The exposed group consisted of 37 cases who developed AKI (Group 1: Exposed-Susceptible, 58.7% of all cases) and 16 controls who remained healthy despite exposure (Group 2: Exposed-Resistant, 6.2% of all controls). An additional 242 unexposed children served as the reference group (Group 3: Unexposed-Healthy, 93.8% of all controls). Twenty-six cases (41.3%) had no documented exposure to the implicated medicines and were excluded from the primary analysis comparing susceptible and resistant children. See Fig 1 for more details.

### Baseline characteristics by group

Table 1 presents baseline characteristics of the three study groups. Exposed-susceptible children were significantly younger than exposed-resistant children (median 17 vs 30 months, p = .003), with notable concentration in infants <12 months (40.5% vs 12.5%). Male sex was more common in susceptible children, though not statistically significant (67.6% vs 43.8%, p = .092). Geographic distribution and socioeconomic indicators showed no significant differences between exposed groups. Anthropometric data were available for limited participants (68% missing); among those with data, weight and height showed trends toward lower values in susceptible children, though small sample sizes precluded definitive conclusions (see Discussion section on ‘Age versus Weight’ for interpretation of null nutritional findings).

**Table 1:** Baseline characteristics of study participants by exposure and outcome status.

Characteristic	Exposed-Susceptible (G1)	Exposed-Resistant (G2)	Unexposed-Healthy (G3)	*p*-value	*p*-value
	(*n* = 37)	(*n* = 16)	(*n* = 242)	(Overall)ᵃ	(G1 vs. G2)ᵇ
**Demographics**					
Age (months)					
Median (*IQR*)	17.0 (11.0–28.0)	30.0 (21.0–48.0)	34.0 (21.0–53.0)	.001	.003
Mean (*SD*)	21.4 (13.8)	35.2 (18.4)	36.5 (20.1)	.001	.005
Age categories					
<12 months	15 (40.5%)	2 (12.5%)	36 (14.9%)	.001	.039
12–23 months	10 (27.0%)	6 (37.5%)	68 (28.1%)		
24–35 months	7 (18.9%)	3 (18.8%)	48 (19.8%)		
≥36 months	5 (13.5%)	5 (31.3%)	90 (37.2%)		
Male sex	25 (67.6%)	7 (43.8%)	126 (52.1%)	.194	.092
Region				.856	.724
WR1	20 (54.1%)	9 (56.3%)	122 (50.4%)		
WR2	7 (18.9%)	2 (12.5%)	62 (25.6%)		
URR	7 (18.9%)	4 (25.0%)	43 (17.8%)		
Other	3 (8.1%)	1 (6.3%)	15 (6.2%)		
Urban residence	28 (75.7%)	11 (68.8%)	184 (76.0%)	.810	.596
**Socioeconomic status**					
Income level				.902	.845
High	28 (75.7%)	13 (81.3%)	178 (73.6%)		
Middle	5 (13.5%)	2 (12.5%)	38 (15.7%)		
Low	4 (10.8%)	1 (6.3%)	26 (10.7%)		
Mother’s education				.594	.802
None/Quranic	13 (35.1%)	7 (43.8%)	115 (47.5%)		
Primary/Secondary	20 (54.1%)	8 (50.0%)	102 (42.1%)		
Tertiary	4 (10.8%)	1 (6.3%)	18 (7.4%)		
Caregiver is mother	25 (67.6%)	12 (75.0%)	202 (83.5%)	.083	.577
**Anthropometric measures**ᶜ					
Weight (kg)					
Median (*IQR*)	9.8 (7.2–11.5)	12.0 (9.5–14.2)	12.6 (9.9–15.8)	.082	.156
*n* with data	11	6	240		
Height (cm)					
Median (*IQR*)	62.0 (59.0–77.0)	75.5 (68.0–88.0)	84.0 (70.0–95.0)	.048	.112
*n* with data	11	6	240		
MUAC (cm)					
Median (*IQR*)	13.5 (12.8–14.8)	14.8 (13.5–15.5)	14.9 (13.6–15.9)	.254	.218
*n* with data	11	6	232		
Malnutrition	2 (5.4%)	1 (6.3%)	9 (3.7%)	.820	.897
**Medical history**					
Prematurity	4 (10.8%)	1 (6.3%)	17 (7.0%)	.732	.589
Prior hospitalization	1 (2.7%)	1 (6.3%)	0 (0.0%)	.003	.545
Prior surgery	3 (8.1%)	1 (6.3%)	0 (0.0%)	<.001	.810

*Note:* G1 = Group 1 (Exposed-Susceptible); G2 = Group 2 (Exposed-Resistant); G3 = Group 3 (Unexposed-Healthy); *IQR* = interquartile range; *SD* = standard deviation; WR1 = Western Region 1; WR2 = Western Region 2; URR = Upper River Region; MUAC = mid-upper arm circumference.

ᵃ*p*-value from Kruskal-Wallis test (continuous variables) or chi-square test (categorical variables) comparing all three groups.

ᵇ*p*-value from Mann-Whitney *U* test (continuous variables) or Fisher’s exact test (categorical variables) comparing only G1 and G2.

ᶜAnthropometric data were missing for the majority of cases; percentages and medians calculated among those with available data.

### Exposure patterns among exposed children

Table 2 presents exposure patterns among the 53 exposed children. A clear dose-response relationship emerged, with susceptible children consuming significantly more contaminated medicines than resistant children (mean 1.8 vs 1.2 medicines, p = .021). Promethazine oral solution showed the strongest association with AKI development (crude OR=3.51, p = .032). Multivitamin supplementation was protective (crude OR=0.29, p = .024), while total medication burden was significantly higher in susceptible children (mean 4.2 vs 2.8 medications, p = .012). See Table 2 for complete exposure details.

**Table 2:** Exposure patterns among children who took Diethylene Glycol-contaminated medicines.

Exposure variable	Exposed-Susceptible (G1)	Exposed-Resistant (G2)	Crude OR	95% CI	*p*-value
	(*n* = 37)	(*n* = 16)			
**Number of adulterated medicines**					
1 medicine	22 (59.5%)	13 (81.3%)	1.00 (ref)	—	—
2 medicines	9 (24.3%)	2 (12.5%)	2.66	[0.51, 14.82]	.236
≥3 medicines	6 (16.2%)	1 (6.3%)	3.55	[0.39, 45.21]	.242
2 + medicines (combined)	15 (40.5%)	3 (18.8%)	2.95	[0.73, 13.22]	.123
Mean ± *SD*	1.8 ± 1.0	1.2 ± 0.4	—	—	.021ᵃ
**Specific medicines taken**					
Promethazine oral solution	20 (54.1%)	4 (25.0%)	3.51	[1.08, 11.84]	.032
Kofexmalin baby cough syrup	9 (24.3%)	3 (18.8%)	1.39	[0.32, 6.38]	.657
MaKOFF baby cough syrup	7 (18.9%)	2 (12.5%)	1.63	[0.29, 9.83]	.584
Magrip N cold syrup	4 (10.8%)	1 (6.3%)	1.82	[0.18, 21.33]	.605
Other brand Promethazine	5 (13.5%)	2 (12.5%)	1.09	[0.18, 6.71]	.925
**Medicine combinations**					
Promethazine only	7 (18.9%)	2 (12.5%)	1.00 (ref)	—	—
Promethazine + ≥1 other	13 (35.1%)	2 (12.5%)	1.86	[0.24, 14.85]	.549
No Promethazine	17 (45.9%)	12 (75.0%)	0.40	[0.07, 2.31]	.302
**Concomitant medications**					
Any concomitant drug	24 (64.9%)	7 (43.8%)	2.38	[0.74, 7.82]	.143
Multivitamin	10 (27.0%)	9 (56.3%)	0.29	[0.09, 0.89]	.024
Acetaminophen	24 (64.9%)	6 (37.5%)	3.06	[0.96, 10.16]	.059
Any antibiotic	8 (21.6%)	3 (18.8%)	1.20	[0.27, 5.47]	.814
Amoxicillin	7 (18.9%)	2 (12.5%)	1.63	[0.29, 9.83]	.584
Gentamicin	1 (2.7%)	0 (0.0%)	—	—	.697ᵇ
Other antibiotics	1 (2.7%)	1 (6.3%)	0.42	[0.02, 7.18]	.550
Anti-inflammatory (any)	28 (75.7%)	9 (56.3%)	2.44	[0.71, 8.88]	.160
Traditional oral medicine	1 (2.7%)	2 (12.5%)	0.19	[0.02, 2.15]	.156
Other cough syrup	13 (35.1%)	5 (31.3%)	1.19	[0.35, 4.14]	.779
**Total medication burden**					
Total unique medications					
Mean ± *SD*	4.2 ± 2.1	2.8 ± 1.3	1.36ᶜ	[1.07, 1.74]	.012ᵃ
0–2 medications	8 (21.6%)	6 (37.5%)	1.00 (ref)	—	—
3–5 medications	18 (48.6%)	9 (56.3%)	1.50	[0.40, 5.76]	.547
≥6 medications	11 (29.7%)	1 (6.3%)	8.25	[0.87, 122.18]	.051

*Note:* G1 = Group 1 (Exposed-Susceptible); G2 = Group 2 (Exposed-Resistant); OR = odds ratio; CI = confidence interval. Odds ratios represent odds of AKI (being in G1 vs. G2) given the exposure.

ᵃ*p*-value from Mann-Whitney *U* test for continuous variables.

ᵇ*p*-value from Fisher’s exact test (cell count = 0).

ᶜOR represents odds per one-unit increase in number of medications.

### Univariable analysis: factors associated with resistance

Table 3 shows the univariable analysis comparing Exposed-Susceptible (Group 1) to Exposed-Resistant (Group 2) children. Several factors were significantly linked to AKI resistance in exposed children (p ≤ .05) or considered for multivariable modeling (p ≤ .20). Age factors had the strongest associations. Each month of age reduced AKI odds (cOR = 0.96 per month, 95% CI [0.93, 0.99], p = .004). Children 36 months or older had much lower risk (cOR = 0.18, 95% CI [0.04, 0.71], p = .009) than younger ones. The protective age effect was non-linear, with greatest vulnerability in the first year. Exposure factors showed dose-response patterns. Taking two or more adulterated medicines (vs. one) increased AKI odds by 3.5 times (cOR = 3.50, 95% CI [1.04, 12.21], p = .041). Promethazine exposure specifically raised AKI odds 3.5 times (cOR = 3.51, 95% CI [1.08, 11.84], p = .032). Total unique medications showed a strong linear association, with each additional medication increasing AKI odds by 36% (cOR = 1.36 per medication, 95% CI [1.07, 1.74], p = .012). Protective medication exposure included multivitamin use, which was associated with 71% reduction in AKI odds (cOR=0.29, 95% CI [0.09, 0.89], p = .024). Sex, geographic region, socioeconomic indicators, and most concomitant medication categories did not show significant associations in univariable analyses.

**Table 3:** Univariable analysis: factors associated with resistance to AKI among exposed children.

Variable	Exposed-Susceptible (G1)	Exposed-Resistant (G2)	Crude OR	95% CI	*p*-value
	(*n* = 37)	(*n* = 16)			
**Demographic factors**					
Age (per month increase)	21.4 ± 13.8	35.2 ± 18.4	0.96	[0.93, 0.99]	.004
Age (per 12-month increase)	—	—	0.64	[0.45, 0.89]	.008
Age ≥ 36 months (vs. < 36)	5 (13.5%)	5 (31.3%)	0.18	[0.04, 0.71]	.009
Age ≥ 24 months (vs. < 24)	12 (32.4%)	8 (50.0%)	0.48	[0.15, 1.51]	.205
Age < 18 months (vs. ≥ 18)	25 (67.6%)	8 (50.0%)	2.08	[0.66, 6.76]	.212
Male sex (vs. female)	25 (67.6%)	7 (43.8%)	2.68	[0.84, 8.83]	.092
Urban residence (vs. rural)	28 (75.7%)	11 (68.8%)	1.42	[0.40, 5.33]	.596
WR1 region (vs. other)	20 (54.1%)	9 (56.3%)	0.92	[0.30, 2.84]	.881
**Socioeconomic factors**					
High income (vs. middle/low)	28 (75.7%)	13 (81.3%)	0.72	[0.17, 3.28]	.665
Mother has formal education	24 (64.9%)	9 (56.3%)	1.44	[0.45, 4.72]	.547
Mother is caregiver	25 (67.6%)	12 (75.0%)	0.70	[0.19, 2.77]	.607
**Exposure factors**					
Number of adulterated medicines					
≥2 medicines (vs. 1)	15 (40.5%)	3 (18.8%)	2.95	[0.73, 13.22]	.123
Per medicine increase	1.8 ± 1.0	1.2 ± 0.4	2.18	[0.90, 5.83]	.085
Promethazine exposure	20 (54.1%)	4 (25.0%)	3.51	[1.08, 11.84]	.032
Kofexmalin exposure	9 (24.3%)	3 (18.8%)	1.39	[0.32, 6.38]	.657
MaKOFF exposure	7 (18.9%)	2 (12.5%)	1.63	[0.29, 9.83]	.584
Magrip exposure	4 (10.8%)	1 (6.3%)	1.82	[0.18, 21.33]	.605
**Medication factors**					
Any concomitant drug	24 (64.9%)	7 (43.8%)	2.38	[0.74, 7.82]	.143
Multivitamin use	10 (27.0%)	9 (56.3%)	0.29	[0.09, 0.89]	.024
Acetaminophen use	24 (64.9%)	6 (37.5%)	3.06	[0.96, 10.16]	.059
Any antibiotic use	8 (21.6%)	3 (18.8%)	1.20	[0.27, 5.47]	.814
Anti-inflammatory use	28 (75.7%)	9 (56.3%)	2.44	[0.71, 8.88]	.160
Traditional medicine use	1 (2.7%)	2 (12.5%)	0.19	[0.02, 2.15]	.156
Total medications (continuous)	4.2 ± 2.1	2.8 ± 1.3	1.36	[1.07, 1.74]	.012
Total medications ≥6 (vs. < 6)	11 (29.7%)	1 (6.3%)	6.36	[0.75, 108.51]	.067
**Nutritional/health factors**ᵃ					
Weight (per kg increase)	9.8 ± 2.3	12.0 ± 3.1	0.78	[0.54, 1.09]	.156
MUAC (per cm increase)	13.8 ± 1.2	14.8 ± 1.3	0.58	[0.27, 1.16]	.142
Malnutrition present	2 (5.4%)	1 (6.3%)	0.86	[0.07, 10.77]	.906
Prematurity	4 (10.8%)	1 (6.3%)	1.83	[0.18, 20.85]	.605

*Note:* G1 = Group 1 (Exposed-Susceptible); G2 = Group 2 (Exposed-Resistant); OR = odds ratio; CI = confidence interval; WR1 = Western Region 1; MUAC = mid-upper arm circumference. Odds ratios represent odds of AKI (being in G1 vs. G2) given the exposure/characteristic. Variables with *p* ≤ .20 (bolded) were considered for inclusion in multivariable models.

ᵃAnthropometric data available for limited sample (G1: *n* = 11; G2: *n* = 6).

### Multivariable analysis: independent protective factors

Table 4 presents result from three multivariable logistic regression models examining independent factors associated with AKI among the 53 exposed children. Variables with p ≤ .20 in univariable screening were considered for inclusion: age (continuous and categorical), sex, number of adulterated medicines, Promethazine exposure, multivitamin use, and total medication burden. Due to multicollinearity between exposure-related variables (number of adulterated medicines, Promethazine exposure, and total medication burden; variance inflation factors >3.5), we constructed three separate models to isolate independent effects while avoiding unstable parameter estimates.

**Table 4:** Multivariable logistic regression models: independent factors associated with AKI among exposed children.

Variable	Model 1: Core Factors		Model 2: Medicine-Specific		Model 3: Total Burden	
	aOR (95% CI)	*p-value*	aOR (95% CI)	*p-value*	aOR (95% CI)	*p-value*
**Age**						
Per 12-month increase	0.58 (0.36–0.92)	.021	0.56 (0.34–0.88)	.014	0.62 (0.40–0.96)	.032
**Sex**						
Male (vs. female)	2.48 (0.71–9.21)	.156	2.85 (0.78–11.04)	.115	2.12 (0.61–7.82)	.241
**Exposure variables**						
≥2 adulterated medicines (vs. 1)	4.21 (1.12–16.85)	.034	—ᵃ	—	—ᵃ	—
Promethazine exposure (yes vs. no)	—ᵃ	—	4.15 (1.15–15.82)	.030	—ᵃ	—
Total unique medications (per med)	—ᵃ	—	—ᵃ	—	1.29 (1.02–1.68)	.037
**Protective factor**						
Multivitamin use (yes vs. no)	0.24 (0.06–0.85)	.028	0.26 (0.07–0.91)	.036	0.28 (0.08–0.95)	.041
**Model statistics**						
*n* (observations)	53		53		53	
Hosmer-Lemeshow χ² (*p*-value)	6.12 (.634)		5.89 (.659)		7.28 (.508)	
AUC (95% CI)	0.82 (0.70–0.95)		0.81 (0.68–0.94)		0.79 (0.66–0.92)	
Pseudo-*R*² (Nagelkerke)	.362		.347		.328	

*Note.* aOR = adjusted odds ratio; CI = confidence interval; AUC = area under the receiver operating characteristic curve. Odds ratios represent odds of acute kidney injury (exposed-susceptible vs. exposed-resistant) adjusting for all other variables in the model.

Model 1 includes age, sex, number of adulterated medicines (dichotomized as ≥2 vs. 1), and multivitamin use.

Model 2 replaces number of medicines with Promethazine-specific exposure while retaining age, sex, and multivitamin use.

Model 3 examines total medication burden (all medication types) as a continuous variable alongside age, sex, and multivitamin use.

All models demonstrated adequate fit based on Hosmer-Lemeshow test (*p* > .05 indicates good fit) and good to excellent discrimination based on AUC (>0.75).

ᵃVariable not included in this model (indicated by em dash).

Model 1 (core protective factors model) included age, sex, number of adulterated medicines, and multivitamin use as predictors. It demonstrated excellent fit (Hosmer-Lemeshow χ² = 6.12, p = .634) and discrimination (ROC curve = 0.82, 95% CI [0.70, 0.95]), explaining 36.2% of AKI variance (Nagelkerke R² = .362). Three factors were protective against AKI. Age showed a strong effect: each additional year reduced AKI odds by 42% (aOR = 0.58, 95% CI [0.36, 0.92], p = .021), suggesting older children have greater resilience to DEG toxicity, likely due to mature renal function. Multivitamin use reduced AKI odds by 76% (aOR = 0.24, 95% CI [0.06, 0.85], p = .028), suggesting a protective mechanism. Exposure to multiple adulterated medicines increased AKI risk, with over a four-fold increase in odds (aOR = 4.21, 95% CI [1.12, 16.85], p = .034), showing a dose-response relationship. Male sex showed a non-significant trend toward increased susceptibility (aOR = 2.48, 95% CI [0.71, 9.21], p = .156), though the wide confidence interval precludes definitive conclusions.

Model 2 replaced the number of medicines variable with Promethazine-specific exposure to assess its impact on toxicity, retaining age, sex, and multivitamin use. This model performed similarly to Model 1 (AUC = 0.81, pseudo-R² = .347) and showed Promethazine exposure quadrupled AKI odds (aOR = 4.15, 95% CI [1.15, 15.82], p = .030), even after controlling for age and multivitamin use. This aligns with lab data showing Promethazine had the highest mean DEG concentration (19.4 mg/mL) among products, suggesting it drove toxicity in this outbreak. The protective effects of age (aOR = 0.56, 95% CI [0.34, 0.88], p = .014) and multivitamin use (aOR = 0.26, 95% CI [0.07, 0.91], p = .036) remained significant and similar to Model 1, demonstrating robustness. Model 3 examined overall pharmaceutical exposure’s influence on AKI risk by including total unique medications as a predictor with age, sex, and multivitamin use. This model showed each additional medication increased AKI odds by 29% (aOR = 1.29 per medication, 95% CI [1.02, 1.68], p = .037), suggesting cumulative toxicity from polypharmacy or that medication count indicated illness severity. However, this model performed slightly worse than Model 1 (AUC = 0.79 vs. 0.82), indicating distinguishing contaminated medicines provided better discrimination. Age and multivitamin effects remained significant (aOR = 0.62, p = .032; aOR = 0.28, p = .041), confirming their protective roles.

Across all three models, consistent age and multivitamin protective effects, with estimates from 0.56-0.62 for age and 0.24-0.28 for multivitamins, indicate these factors independently modify susceptibility to DEG-induced AKI, regardless of exposure operationalization. Model 1 was chosen for its superior discrimination (highest AUC), parsimony, and direct dose-response assessment via the contaminated medicines variable. Full model comparison and goodness-of-fit statistics across all candidate models are reported in Table J in S1 Appendix.

### Dose-response analysis

Table 5 shows the dose-response relationship between the number of DEG-contaminated medicines taken and the probability of developing AKI, adjusted for age and multivitamin use. The relationship was monotonic and statistically significant (test for linear trend, *p* = .018). Compared to taking one contaminated medicine (reference), the adjusted odds ratios (aOR) were as follows: two medicines, aOR = 3.85 (95% CI [1.01, 15.21], *p* = .048) and Three medicines: aOR = 8.72 (95% CI [1.45, 58.33], *p* = .018). Among children taking three contaminated medicines, all developed AKI (6/6, 100%), compared to 63.6% (14/22) of those taking two medicines and 68.0% (17/25) of those taking one medicine. However, even single medicine exposure carried a substantial risk, with two-thirds of exposed children developing AKI.

**Table 5:** Dose-response relationship: number of Diethylene Glycol-contaminated medicines and risk of AKI.

Number of Medicines	Total Exposed	Developed AKI	AKI Rate	Crude OR (95% CI)	Adjusted ORᵃ (95% CI)	*p*-value
1 medicine	25	17	68.0%	1.00 (reference)	1.00 (reference)	—
2 medicines	22	14	63.6%	2.66 (0.51–14.82)	3.85 (1.01–15.21)	.048
3 medicines	6	6	100.0%	3.55 (0.39–45.21)	8.72 (1.45–58.33)	.018
**Test for trend**				*Z* = 2.12, *p* = .034	*Z* = 2.38, *p* = .018	

*Note:* OR = odds ratio; CI = confidence interval; AKI = acute kidney injury.

ᵃAdjusted for age (continuous, per 12 months) and multivitamin use (yes/no) using logistic regression analysis.

### Age-stratified analysis

**Fig 2:**
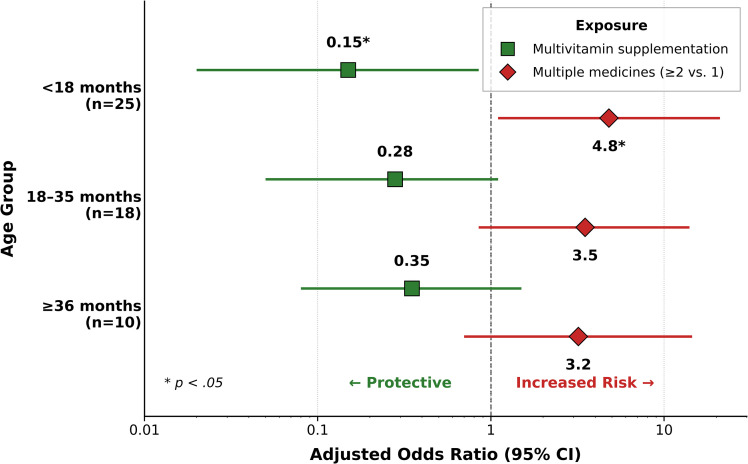
Age-stratified analysis of protective factors against AKI in DEG-Exposed Children. Note: aOR = adjusted odds ratio; CI = confidence interval; asterisks denote statistical significance (*p < .05).

Fig 2 presents the age-stratified analyses examining the effects of protective factors across developmental stages. Children were categorized into three groups: < 18 months (n = 25), 18–35 months (n = 18), and ≥36 months (n = 10). The protective effect of multivitamins appeared strongest in the youngest age group (<18 months: aOR = 0.15, 95% CI [0.02, 0.81], *p* = .018), although confidence intervals overlapped across strata. The dose-response relationship (taking ≥2 vs. 1 medicine) was consistent across age groups, with point estimates ranging from aOR = 3.2 to 4.8, although precision was limited in smaller strata. Among children ≥36 months who took only one contaminated medicine, none developed AKI (0/4), compared to 3/4 who took multiple medicines, suggesting that older children with limited exposure may have been protected by renal maturity.

Forest plot displaying aOR with 95% CIs for protective factors across three age groups: < 18 months (n = 25), 18–35 months (n = 18), and ≥36 months (n = 10). Green squares represent multivitamin supplementation effects, while red diamonds represent the dose-response relationship for consuming ≥2 versus 1 contaminated medicine. The vertical dashed line at aOR = 1.0 indicates no effect. Values are displayed on a logarithmic scale. The protective effect of multivitamin supplementation was strongest in the youngest age group (<18 months: aOR = 0.15, 95% CI [0.02, 0.81], p = .018), although CIs overlapped across strata. The dose-response relationship showed consistent point estimates across age groups (aOR range: 3.2 to 4.8), with limited precision in smaller strata. Among children ≥36 months who consumed only one contaminated medicine, none developed AKI (0/4), compared to 75% (3/4) who consumed multiple medicines, suggesting that older children with limited exposure may have been protected by renal maturity. All estimates were derived from multivariable logistic regression models adjusted for region, sex, and clinical covariates.

### Sensitivity analyses

Propensity Score-Matched analysis (Table C in S1 Appendix): We conducted a propensity score-matched analysis to address confounding, pairing exposed susceptible and resistant children by age, sex, region, and income. Among 14 matched pairs (2 resistant children unmatched), results aligned with primary analyses: multivitamin use (matched OR = 0.21, 95% CI [0.04, 0.92], p = .041) and taking ≥2 medicines (matched OR = 4.50, 95% CI [1.02, 21.15], p = .047) remained significant. Multiple imputation for missing data (Table D in S1 Appendix): We performed multiple imputations (20) for anthropometric variables (60–80% missing). Results were qualitatively similar to complete case analyses, with weight-for-age z-score showing a non-significant protective trend (pooled aOR = 0.72 per SD increase, 95% CI [0.44, 1.18], p = .196), suggesting nutritional status may contribute to susceptibility but was underpowered. Alternative exposure definitions (Table E in S1 Appendix): Redefining exposure to any documented use of individual contaminated medicines classified 41 cases (65.1% of all) as exposed. In this expanded group, primary findings remained: age (aOR = 0.61 per year, p = .031), multivitamins (aOR = 0.28, p = .035), and dose-response (aOR = 3.88 for ≥2 medicines, p = .041).

### Three-group comparison: contextualizing the resistant phenotype

To compare resistant children to unexposed healthy controls, we conducted a multinomial logistic regression with three outcomes: unexposed-healthy (reference), exposed-resistant, and exposed-susceptible (Table A in S1 Appendix). Exposed-Resistant children were likelier to be younger (RRR = 0.98 per month, p = .041) and from urban areas (RRR = 2.15, p = .082), indicating similar exposure opportunities as susceptible children but with other protective factors. The main difference between Exposed-Resistant and Exposed-Susceptible children (comparing the RRRs) was multivitamin use and the number of medicines, highlighting these factors’ impact on outcomes among the exposed.

### Unexplained cases without documented exposure

Twenty-six cases (41.3% of all AKI cases) had no documented exposure to the four implicated contaminated medicines. Supplementary analyses (Table B in S1 Appendix) revealed that these children were similar in age to exposed cases (median = 19 months vs. 17 months, *p* = .681) but had higher rates of “other cough syrup” use (46.2% vs. 35.1%, *p* = .378) and traditional oral medicines (7.7% vs. 2.7%, *p* = .298), although the differences were not statistically significant. This pattern suggests potential exposure misclassification (underreporting of contaminated medicine use) or contamination of additional unreported products. These cases are discussed further in the limitations section of this paper.

## Discussion

This case-case-control study is the first to investigate protective factors against AKI in children exposed to DEG-contaminated medicines. Among 53 children with documented exposure to adulterated pediatric syrups during The Gambia’s 2022 outbreak, 16 (30.2%) did not develop AKI despite confirmed ingestion of contaminated products. We identified three independent protective factors: older age (adjusted OR = 0.58 per year, 95% CI [0.36, 0.92]), multivitamin supplementation (aOR = 0.24, 95% CI [0.06, 0.85]), and limiting exposure to a single contaminated medicine (aOR = 4.21 for ≥2 vs. 1 medicine, 95% CI [1.12, 16.85]). These findings suggest potential modifiable factors that are associated with reduced DEG toxicity and offer actionable insights for clinical management of exposed children in future outbreaks.

### Age versus weight: developmental maturity supersedes body size

An initially counterintuitive finding merits explicit discussion: weight and nutritional status showed no significant association with AKI risk among exposed children, despite theoretical expectations that smaller children would receive higher mg/kg doses and experience different volumes of distribution. This null finding has important mechanistic implications and aligns with our understanding of DEG toxicodynamics. Our data suggest that developmental maturity, rather than body size per se, is the primary determinant of susceptibility. This is evidenced by several observations: (1) Age remained the strongest protective factor (aOR=0.58 per year) even after controlling for weight in imputed analyses; (2) Within age strata, weight showed inconsistent associations; (3) The protective effect of age was most pronounced in comparing infants <18 months to older children, a developmental transition marked by renal functional maturation rather than proportional weight gain.

This pattern reflects that DEG nephrotoxicity is driven primarily by renal capacity for metabolite excretion and cellular repair, which are developmental phenomena not strictly proportional to body size. Younger children have: (1) immature glomerular filtration and tubular secretion limiting toxic metabolite elimination [15]; (2) incomplete nephron development and reduced renal reserve compromising injury recovery [41]; (3) evolving drug-metabolizing enzyme systems affecting DEG pharmacokinetics [51]. Supporting this interpretation, previous DEG outbreaks have similarly shown stronger age-stratified than weight-stratified risk patterns. In Panama’s 2006 outbreak, children <2 years had 72% case-fatality compared to 41% in older children despite similar weight-for-age distributions [12]. Indonesia’s 2022 outbreak showed median case age of 23 months but no reported weight associations [11]. The lack of malnutrition association may also reflect that the 68% missingness of anthropometric data limited our power to detect effects. However, multiple imputation analyses (Sensitivity Analysis 2; Table D in S1 Appendix) yielded similar null findings, suggesting the effect, if present, is small relative to developmental age.

### Age-dependent vulnerability to diethylene glycol toxicity

The profound age-dependent susceptibility observed in our study aligns with fundamental principles of developmental toxicology and pediatric pharmacology. Each additional year of age conferred a 42% reduction in AKI odds among exposed children, with the effect being most pronounced in infants under 18 months. This pattern reflects multiple age-related factors that influence DEG toxicokinetics and toxicodynamics. First, younger children have immature alcohol dehydrogenase and aldehyde dehydrogenase enzyme systems that are responsible for metabolizing DEG to its nephrotoxic metabolite, diglycolic acid (DGA) [2,8,52]. Paradoxically, while slower metabolism might seem protective, the prolonged parent compound half-life in young children is associated with an extended window for oxidative metabolism, ultimately generating more DGA over time [51]. Second, immature renal function in infants, characterized by lower glomerular filtration rates, reduced tubular secretory capacity, and incomplete nephron maturation, compromises the kidneys’ ability to excrete toxic metabolites and repair cellular injury [15,41]. Third, higher metabolic rates and greater fluid turnover in younger children may result in higher tissue concentrations of DEG relative to body weight when equivalent doses are administered [16].

Our findings corroborate the age patterns observed in previous DEG outbreaks. During Panama’s 2006 epidemic, children under 2 years had a case fatality rate of 72% compared to 41% in older children, although formal age-stratified susceptibility analyses were not reported [12]. Similarly, Indonesia’s 2022 outbreak predominantly affected children under 5 years, with median age of cases at 23 months [53]. The consistency across outbreaks, despite differences in DEG concentrations, co-exposures, and healthcare access, suggests that developmental vulnerability is an intrinsic biological phenomenon rather than a context-specific artifact. In terms of clinical implications, following any suspected DEG exposure, infants and toddlers require intensive surveillance and early nephrology consultation, even in the absence of immediate symptoms. Age-stratified triage protocols could optimize resource allocation in outbreak settings where healthcare capacity is strained.

### Multivitamin supplementation: observed association and confounding considerations

The observed protective association of multivitamin use (aOR = 0.24, 95% CI: 0.06–0.85) represents a potentially important finding that nonetheless requires cautious interpretation owing to the possibility of unmeasured confounding. Several biological mechanisms could plausibly account for a true protective effect: antioxidants such as vitamins C and E, selenium, and zinc may mitigate DEG-induced oxidative stress [54–58]; B vitamins support mitochondrial function during toxic insults [59]; and vitamin B6 participates in oxalate metabolism, a pathway relevant to DEG nephrotoxicity [60]. Several lines of evidence lend support to a biological mechanism, including the persistence of the protective effect after propensity score matching that adjusted for measured socioeconomic indicators, the observation that the effect was strongest among the youngest children (aged less than 18 months) in a pattern consistent with developmental vulnerability to oxidative stress, and the dose-response relationship between antioxidant intake and nephroprotection reported in other toxicity models [60]. However, multivitamin use may also serve as a proxy for socioeconomic status, parental health literacy, or general health-protective behaviors that independently reduce AKI risk through non-vitamin mechanisms. Indeed, multivitamin users had somewhat higher maternal education levels, and we cannot rule out the influence of unmeasured healthcare-seeking behaviors or other protective practices within multivitamin-using families. To address this concern, we adjusted for measured socioeconomic indicators including income score, maternal education, and household assets; conducted propensity score matching on age, sex, region, and income, which yielded a matched adjusted odds ratio of 0.21 that remained statistically significant; examined effect consistency across socioeconomic strata; and noted that the protective effect was strongest in the youngest children (aOR = 0.15 for those under 18 months), a pattern less consistent with socioeconomic confounding than with a biological mechanism.

Despite these analytic efforts, we cannot definitively establish causality for the multivitamin-AKI association, as parental health literacy beyond maternal education, healthcare-seeking behaviors, quality of supportive care received, and concurrent unmeasured protective behaviors remain potential confounders that could not be addressed with available data. The observed effect may therefore reflect true biological protection via antioxidant mechanisms, residual confounding by unmeasured socioeconomic and health literacy factors, or a combination of both operating simultaneously. Given this uncertainty, we present multivitamin use as an observed association rather than a proven protective factor and explicitly emphasize that these observational data alone do not warrant clinical implementation. If validated in randomized trials, prophylactic antioxidant supplementation could be considered for children exposed to DEG-contaminated products; however, such trials must measure and adjust for health literacy and socioeconomic confounders, use standardized antioxidant formulations, include biomarkers of oxidative stress, and assess dose-response relationships before any recommendation can be made. Until such evidence is available, multivitamin supplementation should never substitute for definitive supportive care, including hydration and fomepizole administration [61].

### Dose-response relationship and medicine-specific toxicity

The clear dose-response relationship observed in this study, with the odds of AKI increasing 4.2-fold when taking two or more contaminated medicines compared to one, supports a causal interpretation according to Bradford Hill criteria but does not establish causation in this observational design [24]. This monotonic relationship persisted across sensitivity analyses and age strata, and the test for linear trend was statistically significant (p = .018). Among children exposed to three contaminated medicines, 100% developed AKI (6/6), compared to 63.6% (14/22) with two medicines and 68.0% (17/25) with one medicine. Even single-drug exposure carried substantial risk, underscoring that any DEG exposure is dangerous and no threshold can be considered safe in the pediatric population.

The medicine-specific analysis (Table F in S1 Appendix) revealed that promethazine oral solution had the strongest toxicity (aOR = 4.15, 95% CI [1.15, 15.82]), consistent with laboratory data showing that this product had the highest mean DEG concentration (19.4 mg/mL) among tested samples. The correlation between adjusted odds ratios and DEG concentrations across the four medicines (Spearman ρ = 0.89), although not statistically significant with only four data points, supports a direct relationship between contamination level and clinical toxicity. This finding validates the utility of medicine-specific risk assessments in outbreak investigations and highlights the importance of batch-level testing in guiding clinical triage. Previous outbreaks have similarly demonstrated dose-dependent toxicity. In Haiti’s 1995–1996 epidemic, children who consumed larger volumes of contaminated acetaminophen syrup had higher mortality rates [62], and Panama’s outbreak showed that prolonged use (>7 days) substantially increased risk [12]. The convergence of evidence across multiple outbreaks, populations, and contamination scenarios reinforces that cumulative DEG dose, whether from multiple medicines, higher-concentration products, longer duration of use, or higher per-dose volumes, is the primary determinant of toxicity.

### The unexplained cases: implications for outbreak investigation

A substantial proportion of AKI cases (26/63, 41.3%) lacked documented exposure to the four implicated drugs. Our analyses revealed these “unexplained” cases had similar demographics, symptoms, and outcomes to exposed cases but significantly higher use of unspecified “other cough syrups” (46.2% vs. 12.0% in unexposed controls, p < .001). Several interpretations exist for this finding. First, exposure misclassification due to recall bias or inability to specify brand names may have led to underreporting of implicated medicines. The traumatic nature of child illness and death may have impaired caregivers’ ability to accurately remember product names months later. Second, additional contaminated products beyond the four identified medicines may have circulated undetected. The Medicines Control Agency of The Gambia tested products brought by families but could not test medicines that were discarded, consumed, or never reported by families. Third, batch-specific variations in DEG concentration could have resulted in some batches being falsely labeled as “safe” if testing was performed on uncontaminated samples from the same product line. The finding that 16 exposed children did not develop AKI, while 26 unexposed children did, creates an apparent paradox that demands explanation. If the four medicines were truly the sole cause of the outbreak, one would expect a near-perfect correspondence between exposure and disease. This imperfect correlation suggests either widespread exposure misclassification or multiple contaminated products in circulation. From a public health perspective, this underscores the critical importance of comprehensive medicine screening during outbreak investigations, including testing of “other” or generic products that may not initially raise suspicion.

### Clinical and public health implications

Our findings have immediate practical applications for managing DEG exposure in future outbreaks, which remain common [3,20,53,63]. First, age-based risk stratification should prioritize intensive monitoring and early intervention for children under 2 years, who face disproportionate risk. Second, aggressive supportive care, including hyperhydration protocols to promote toxin elimination and fomepizole administration to block alcohol dehydrogenase, should be initiated at the first suspicion of DEG exposure, particularly in young children or those with polypharmacy exposure [61,64]. Third, prophylactic antioxidant supplementation should be considered in exposed children, although this requires prospective validation. Fourth, a careful medication history must document not only whether the implicated medicines were taken but also the number of different products, duration of use, and estimated total volume consumed, as these factors modulate individual risk. From a prevention standpoint, these findings reinforce the urgent need to strengthen pharmaceutical regulatory systems in LMICs [22,65]. The recurrence of DEG contamination - with major outbreaks in Nigeria (1990) [3,65], Bangladesh (1992) [4], Haiti (1995–1996) [62], Panama (2006) [12], India (2019–2020) [53], Indonesia (2022) [53], Uzbekistan (2022) [20], The Gambia (2022), and Cameroon (2023) [20] - demonstrates systemic failures in medicine quality assurance. Import screening, batch testing, supply chain traceability, and severe penalties for adulteration are essential but are often poorly implemented or enforced in resource-constrained settings [21,22]. The WHO’s 2023 recommendations for strengthening substandard and falsified medical product surveillance [63] provide a framework, but implementation requires sustained political will and international support.

### Strengths and limitations

This study had several notable strengths. The case-case-control design, which compares exposed children with different outcomes, provides more robust causal inference than traditional case-control studies and identifies effect modifiers more efficiently than full cohort designs [25]. The comprehensive exposure assessment captured specific medicines, combinations, concomitant medications, and dose proxies, enabling a nuanced characterization of the exposure landscape. Rigorous statistical methods, including propensity score matching, multiple imputation, and multiple sensitivity analyses, were employed to demonstrate the robustness of the findings. Furthermore, the investigation was conducted in close collaboration with national and international health authorities, ensuring both data quality and policy relevance.

Our case definition included both confirmed cases (92.1%) with laboratory or clinical confirmation and probable cases (7.9%, n = 5) who died before laboratory testing could be performed. While this inclusive approach was necessary for outbreak surveillance and served to avoid survivor bias, it introduces the potential for misclassification, as some probable cases may have had alternative causes of death unrelated to DEG-induced AKI. However, several factors suggest that such misclassification is minimal. All probable cases had documented DEG exposure and presented during the outbreak period with symptom profiles identical to those of confirmed cases, and the Gambian Ministry of Health’s causality assessment attributed these deaths to contaminated medicines on the basis of temporal, clinical, and epidemiological evidence. Moreover, any misclassification would bias findings toward the null by reducing the ability to detect protective factors, rendering our results conservative estimates. If non-AKI deaths were misclassified as probable AKI cases, they would likely be distributed randomly between the susceptible and resistant groups, attenuating observed associations rather than creating spurious ones. Thus, the reported associations for age, multivitamins, and dose-response relationships, if anything, underestimate the true protective effects.

Several important limitations must also be acknowledged. First, the sample size of the resistant group (n = 16) limited statistical power to detect moderate effect sizes and precluded certain subgroup analyses, resulting in wide confidence intervals and potentially underpowered non-significant trends such as those observed for sex differences and acetaminophen effects. Among 53 children with documented DEG exposure, 30.2% (n = 16) remained healthy, yielding a 70% attack rate consistent with other outbreaks but constraining statistical inference. The limited size of the resistant group reflects the high inherent toxicity of DEG at the concentrations encountered (7.1–24.8 mg/mL), the young age and baseline vulnerability of the population (median 19 months), the outbreak investigation’s focus on symptomatic children which may have led to under-ascertainment of asymptomatic exposed children, a three- to six-month enrollment delay that may have missed transient mild cases, and the possibility that some resistant children experienced undetected subclinical AKI that resolved spontaneously. Consequently, the study had 80% power only for large effects (OR ≥ 3.0), stratified analyses yielded very small cells (n = 2–4), individual observations exerted disproportionate influence on estimates, and potential interactions such as those between age and multivitamin use or age and dose may have gone undetected. Despite these constraints, the groups were well balanced on measured confounders after propensity score matching, the main findings exhibited large effect sizes that persisted across sensitivity analyses, the dose-response relationship demonstrated perfect monotonicity, and biological plausibility was supported by prior outbreak data. A stronger design would have enrolled at least 50 resistant children through active surveillance, prospective enrollment, or multi-outbreak pooling.

The wide confidence intervals in key findings require careful interpretation. For instance, the adjusted odds ratio for multivitamin use was 0.24 (95% CI: 0.06–0.85), spanning an eight-fold range, while the dose-response estimate for two or more medicines was 4.21 (95% CI: 1.12–16.85), spanning a fifteen-fold range, and the estimate for sex was 2.48 (95% CI: 0.71–9.21), which included the null. These wide intervals reflect sampling variability rather than systematic error; point estimates remain unbiased despite imprecision. Notably, statistically significant findings are robust as effects with p < .05 had sufficiently large effect sizes to overcome imprecision, while non-significant trends like sex differences may represent true moderate effects that were underpowered or may genuinely be null. Consistency across all three multivariable models, which yielded similar point estimates for age (0.56–0.62) and multivitamins (0.24–0.28), further strengthens the inferential basis. Post-hoc power calculations indicated 80% power to detect OR ≥ 3.0 for exposures with 50% versus 20% prevalence, confirming the study was underpowered for moderate effects in the range of OR 1.5–2.5. Accordingly, we emphasize large, clinically meaningful effects that achieved statistical significance despite limited power, treat moderate effects as hypothesis-generating, and note that precise quantification of all associations requires replication in larger samples.

Second, exposure assessment relied on caregiver recall three to six months post-outbreak, introducing variable-specific recall bias affecting findings. Variables most susceptible to this bias include specific medicine brands, which caregivers may have confused or forgotten, and concomitant medication details like timing, dosing, and duration. For medicine brands, non-differential misclassification would bias estimates toward the null, underestimating the true protective effect of limiting exposure, while differential misclassification if bereaved caregivers of cases recalled exposures more intensively would overestimate exposure-AKI associations, though consistent dose-response patterns across outbreaks argue against substantial differential bias. For concomitant medications, recall uncertainty would primarily affect precision rather than association direction. Multivitamin use is also prone to recall error, as parents may have misremembered whether vitamins were given during the outbreak versus habitually; non-differential misclassification would bias this protective association toward the null, suggesting that the observed adjusted odds ratio of 0.24 may underestimate the true effect. In contrast, variables least susceptible to recall bias include age and demographic data, verified against health cards when available, serious outcomes like hospitalization and death, and DEG exposure itself, for which recall was enhanced by the outbreak investigation, media coverage, and visual aids depicting contaminated medicine bottles. Mitigating strategies included temporal anchoring using the culturally salient Tobaski festival and standardized probing across all groups. The net impact of recall bias is primarily non-differential misclassification that biases estimate conservatively toward the null, and the main findings remained robust across propensity score matching and sensitivity analyses.

Third, we lacked data on precise dosing parameters, including volume per administration, frequency, and duration, which would have enabled more refined dose-response modeling. Fourth, anthropometric data were missing for most participants (68%), limiting the assessment of nutritional status as a protective factor, although imputation analyses suggested that this missingness did not substantially affect the conclusions. Fifth, we were unable to measure genetic factors, such as alcohol dehydrogenase polymorphisms, that may influence individual susceptibility to DEG toxicity [66]. Finally, the findings are specific to this outbreak context pediatric syrups with particular DEG concentrations in a Gambian population and generalizability to other settings, formulations, or populations requires confirmation.

### Future research directions

Several critical research priorities have emerged from this study. First, prospective cohort studies of exposed children (identified through outbreak surveillance or product recalls) are needed to validate the protective factors and inform evidence-based interventions. Such studies should include biomarker measurements (serum DEG, DGA, and glycolate levels) to objectively quantify exposure and enable pharmacokinetic modeling. Second, randomized controlled trials evaluating prophylactic antioxidant supplementation in exposed children could establish whether the observed association with multivitamins is causal and clinically beneficial. Such trials would be ethically justifiable, given the safety of multivitamins and the absence of proven prophylactic treatments beyond fomepizole. Third, genetic studies examining polymorphisms in alcohol dehydrogenase, aldehyde dehydrogenase, and renal transporter genes could identify biomarkers for individual susceptibility and inform targeted screening. Fourth, animal models incorporating age-dependent toxicokinetics and antioxidant interventions could elucidate the biological mechanisms and optimize protective strategies. Finally, implementation research evaluating the real-world application of age-based risk stratification protocols and early intervention strategies would assess their feasibility and effectiveness in resource-limited settings where future outbreaks are most likely.

## Conclusions

This study demonstrates that substantial heterogeneity exists in susceptibility to DEG-induced AKI among exposed children, with 30% remaining healthy despite the documented consumption of contaminated medicines. Younger age, polypharmacy with multiple adulterated products, and absence of multivitamin supplementation were independently associated with increased risk, while older children taking a single contaminated medicine who received multivitamins were relatively protected. These findings identify high-risk subgroups requiring intensive surveillance and suggest potential prophylactic strategies that warrant further validation. As DEG contamination continues to cause pediatric deaths globally despite decades of awareness, understanding the determinants of differential susceptibility is essential for optimizing clinical management and may ultimately save lives in future outbreaks. Equally urgent is the need for systemic strengthening of pharmaceutical quality assurance systems to prevent these avoidable tragedies.

## Supporting information

S1 Appendix Supplementary tables. Table A. Multinomial logistic regression: three-group comparison contextualizing resistant children. Table B. Characteristics and exposure patterns of cases without documented adulterated medicine exposure. Table C. Propensity score-matched analysis: protective factors among exposed children. Table D. Sensitivity analysis using multiple imputation for missing anthropometric data. Table E. Sensitivity analysis: results under alternative exposure definitions. Table F. Medicine-specific toxicity: individual medicine effects on acute kidney injury risk. Table G. Interaction effects: age and multivitamin moderation of medicine toxicity. Table H. Geographic distribution and clustering analysis. Table I. Complete univariable analysis: all variables examined. Table J. Model comparison and goodness-of-fit statistics.
(DOCX)

## References

[1] Geiling EMK. Pathologic effects of elixir of sulfanilamide (diethylene glycol) poisoning: a clinical and experimental correlation: final report. JAMA. 1938;111:919. doi: 10.1001/jama.1938.72790360005007

[2] Schep LJ, Slaughter RJ, Temple WA, Beasley DMG. Diethylene glycol poisoning. Clin Toxicol (Phila). 2009;47(6):525–35. doi: 10.1080/15563650903086444 19586352

[3] Okuonghae HO, Ighogboja IS, Lawson JO, Nwana EJ. Diethylene glycol poisoning in Nigerian children. Ann Trop Paediatr. 1992;12(3):235–8. doi: 10.1080/02724936.1992.11747577 1280035

[4] Hanif M, Mobarak MR, Ronan A, Rahman D, Donovan JJ Jr, Bennish ML. Fatal renal failure caused by diethylene glycol in paracetamol elixir: the Bangladesh epidemic. BMJ. 1995;311(6997):88–91. doi: 10.1136/bmj.311.6997.88 7613408 PMC2550149

[5] Alkahtani S, Sammons H, Choonara I. Epidemics of acute renal failure in children (diethylene glycol toxicity). Arch Dis Child. 2010;95(12):1062–4. doi: 10.1136/adc.2010.183392 21062849

[6] McMartin KE, Wallace KB. Calcium oxalate monohydrate, a metabolite of ethylene glycol, is toxic for rat renal mitochondrial function. Toxicol Sci. 2005;84(1):195–200. doi: 10.1093/toxsci/kfi062 15601675

[7] Ferguson SA, Law CD Jr, Abshire JS. Developmental treatment with bisphenol A or ethinyl estradiol causes few alterations on early preweaning measures. Toxicol Sci. 2011;124(1):149–60. doi: 10.1093/toxsci/kfr201 21813462

[8] Borron SW, Baud FJ, Garnier R. Intravenous 4-methylpyrazole as an antidote for diethylene glycol and triethylene glycol poisoning: a case report. Vet Hum Toxicol. 1997;39(1):26–8. 9004463

[9] Bastani P. Acute kidney injury among children likely associated with diethylene glycol–contaminated medications - The Gambia, June-September 2022. MMWR. 2023;72:217–22. 36862590 10.15585/mmwr.mm7209a1 PMC9997663

[10] Bittaye M, Byakika-Tusiime J, Adisso L, Pavlin BI, Muteba M, Jammeh AH. Causes and risk factors for an acute kidney injury outbreak among children in The Gambia, June – September 2022: A case-cohort study. PLoS One. 2025;20(e0324931). doi: 10.1371/journal.pone.0324931 PMC12161566 40504873

[11] Umar TP, Jain N, Azis H. Endemic rise in cases of acute kidney injury in children in Indonesia and Gambia: what is the likely culprit and why?. Kidney Int. 2023;103(3):444–7. doi: 10.1016/j.kint.2022.12.004 36639266

[12] Rentz ED, Lewis L, Mujica OJ, Barr DB, Schier JG, Weerasekera G, et al. Outbreak of acute renal failure in Panama in 2006: a case-control study. Bull World Health Organ. 2008;86(10):749–56. doi: 10.2471/blt.07.049965 18949211 PMC2649516

[13] Newton PN, Caillet C, Guerin PJ. A link between poor quality antimalarials and malaria drug resistance?. Expert Review of Anti-infective Therapy. 2016;14:531–3. doi: 10.1080/14787210.2016.1187560 27187060

[14] World Health Organization. Substandard and falsified medical products. https://www.who.int/news-room/fact-sheets/detail/substandard-and-falsified-medical-products 2024. Accessed 2025 October 18.

[15] Hinchliffe SA, Sargent PH, Howard CV, Chan YF, van Velzen D. Human intrauterine renal growth expressed in absolute number of glomeruli assessed by the disector method and Cavalieri principle. Lab Invest. 1991;64(6):777–84. 2046329

[16] Barr J, Brenner-Zada G, Heiman E, Pareth G, Bulkowstein M, Greenberg R, et al. Unlicensed and off-label medication use in a neonatal intensive care unit: a prospective study. Am J Perinatol. 2002;19(2):67–72. doi: 10.1055/s-2002-23557 11938479

[17] Luyckx VA, Miljeteig I, Ejigu AM, Moosa MR. Ethical Challenges in the Provision of Dialysis in Resource-Constrained Environments. Semin Nephrol. 2017;37(3):273–86. doi: 10.1016/j.semnephrol.2017.02.007 28532556

[18] World Health Organization. Safe preparation, storage and handling of powdered infant formula: guidelines. Geneva: World Health Organization. 2007.

[19] World Health Organization. WHO urges action to protect children from contaminated medicines. https://www.who.int/news/item/23-01-2023-who-urges-action-to-protect-children-from-contaminated-medicines 2023. Accessed 2025 October 18.

[20] World Health Organization. Medical Product Alert N°5/2023: Substandard (contaminated) syrup medicines. https://www.who.int/news/item/19-07-2023-medical-product-alert-n-5-2023--substandard-(contaminated)-syrup-medicines 2023. Accessed 2025 October 18.

[21] Board on Global Health. Countering the Problem of Falsified and Substandard Drugs. Washington, D.C.: National Academies Press. 2013. doi: 10.17226/18272 24872973

[22] Newton PN, Bond KC, 53 signatories from 20 countries. COVID-19 and risks to the supply and quality of tests, drugs, and vaccines. Lancet Glob Health. 2020;8(6):e754–5. doi: 10.1016/S2214-109X(20)30136-4 32278364 PMC7158941

[23] Ministry of Health. Causality Assessment Report on Acute Kidney Injury Outbreak in Children, June–October 2022. Banjul, The Gambia: Ministry of Health. 2022.

[24] Hill AB. The Environment and Disease: Association or Causation? Proceedings of the Royal Society of Medicine. 1965;58:295–300. doi: 10.1177/003591576505800503 14283879 PMC1898525

[25] O’Brien KM, Lawrence KG, Keil AP. The Case for Case-Cohort: An Applied Epidemiologist’s Guide to Reframing Case-Cohort Studies to Improve Usability and Flexibility. Epidemiology. 2022;33(3):354–61. doi: 10.1097/EDE.0000000000001469 35383643 PMC9172927

[26] Rothman KJ, Lash TL, VanderWeele TJ, Haneuse S. Modern epidemiology. 4th ed. Philadelphia: Wolters Kluwer / Lippincott Williams & Wilkins. 2021.

[27] Goldstein L, Langholz B. Risk set sampling in epidemiologic cohort studies. Statist Sci. 1996. doi: 10.1214/ss/1032209663

[28] Gambia Bureau of Statistics, ICF. The Gambia Demographic and Health Survey 2019-20. Banjul, The Gambia and Rockville, Maryland, USA: GBoS and ICF. 2021.

[29] Barrow A, Bittaye M, Bittaye SO, Oguche SM. Clinical phenotypes and severity stratification in pediatric diethylene glycol poisoning: a latent class analysis of the Gambia acute kidney injury outbreak. Pediatr Nephrol. 2026;41(6):1851–65. doi: 10.1007/s00467-026-07148-2 41580573

[30] World Health Organization. WHO Outbreak Communication Planning Guide. 2008. https://www.afro.who.int/sites/default/files/2017-06/outbreak_com_plan_guide.pdf

[31] KDIGO. KDIGO clinical practice guideline for acute kidney injury. Kidney Int Suppl. 2012;2:1–138. doi: 10.1038/kisup.2012.1

[32] Akcan-Arikan A, Zappitelli M, Loftis LL, Washburn KK, Jefferson LS, Goldstein SL. Modified RIFLE criteria in critically ill children with acute kidney injury. Kidney Int. 2007;71(10):1028–35. doi: 10.1038/sj.ki.5002231 17396113

[33] Hosmer DW, Lemeshow S, Sturdivant RX. Applied logistic regression. 3 ed. Hoboken, New Jersey: Wiley. 2013.

[34] Bursac Z, Gauss CH, Williams DK, Hosmer DW. Purposeful selection of variables in logistic regression. Source Code Biol Med. 2008;3:17. doi: 10.1186/1751-0473-3-17 19087314 PMC2633005

[35] O’brien RM. A caution regarding rules of thumb for variance inflation factors. Qual Quant. 2007;41:673–90. doi: 10.1007/s11135-006-9018-6

[36] Durrleman S, Simon R. Flexible regression models with cubic splines. Stat Med. 1989;8(5):551–61. doi: 10.1002/sim.4780080504 2657958

[37] Hosmer DW, Lemesbow S. Goodness of fit tests for the multiple logistic regression model. Communications in Statistics - Theory and Methods. 1980;9(10):1043–69. doi: 10.1080/03610928008827941

[38] Hanley JA, McNeil BJ. The meaning and use of the area under a receiver operating characteristic (ROC) curve. Radiology. 1982;143(1):29–36. doi: 10.1148/radiology.143.1.7063747 7063747

[39] Akaike H. A new look at the statistical model identification. IEEE Trans Automat Contr. 1974;19(6):716–23. doi: 10.1109/tac.1974.1100705

[40] Cuzick J. A Wilcoxon-type test for trend. Stat Med. 1985;4(1):87–90. doi: 10.1002/sim.4780040112 3992076

[41] Bruel A, Rozé J-C, Quere M-P, Flamant C, Boivin M, Roussey-Kesler G, et al. Renal outcome in children born preterm with neonatal acute renal failure: IRENEO-a prospective controlled study. Pediatr Nephrol. 2016;31(12):2365–73. doi: 10.1007/s00467-016-3444-z 27335060

[42] Rosenbaum PR, Rubin DB. The central role of the propensity score in observational studies for causal effects. Biometrika. 1983;70(1):41–55. doi: 10.1093/biomet/70.1.41

[43] Austin PC. Optimal caliper widths for propensity-score matching when estimating differences in means and differences in proportions in observational studies. Pharm Stat. 2011;10(2):150–61. doi: 10.1002/pst.433 20925139 PMC3120982

[44] Austin PC. Balance diagnostics for comparing the distribution of baseline covariates between treatment groups in propensity-score matched samples. Stat Med. 2009;28(25):3083–107. doi: 10.1002/sim.3697 19757444 PMC3472075

[45] Buuren SV, Groothuis-Oudshoorn K. Mice: Multivariate imputation by chained equations in R. J Stat Soft. 2011;45. doi: 10.18637/jss.v045.i03

[46] Rubin DB. Multiple Imputation for Nonresponse in Surveys. 1st ed. Wiley. 1987. doi: 10.1002/9780470316696

[47] Moran PAP. Notes on Continuous Stochastic Phenomena. Biometrika. 1950;37:17–23. doi: 10.1093/biomet/37.1-2.17 15420245

[48] Little RJA. A Test of Missing Completely at Random for Multivariate Data with Missing Values. Journal of the American Statistical Association. 1988;83(404):1198–202. doi: 10.1080/01621459.1988.10478722

[49] World Medical Association. World Medical Association Declaration of Helsinki: Ethical Principles for Medical Research Involving Human Subjects. JAMA. 2013;310:2191. doi: 10.1001/jama.2013.281053 24141714

[50] von Elm E, Altman DG, Egger M, Pocock SJ, Gøtzsche PC, Vandenbroucke JP, et al. The Strengthening the Reporting of Observational Studies in Epidemiology (STROBE) Statement: guidelines for reporting observational studies. Int J Surg. 2014;12(12):1495–9. doi: 10.1016/j.ijsu.2014.07.013 25046131

[51] Lu H, Rosenbaum S. Developmental pharmacokinetics in pediatric populations. The Journal of Pediatric Pharmacology and Therapeutics. 2014;19:262–76. doi: 10.5863/1551-6776-19.4.262 25762871 PMC4341411

[52] Perazella MA. Hiding in Plain Sight: Catastrophic Diethylene Glycol Poisoning in Children. Kidney360. 2023;4(11):1534–5. doi: 10.34067/KID.0000000000000269 37831818 PMC10695641

[53] World Health Organization. Medical Product Alert N°6/2022: Substandard (contaminated) paediatric medicines. 2022. https://www.who.int/news/item/05-10-2022-medical-product-alert-n-6-2022-substandard-(contaminated)-paediatric-medicines

[54] Tapia E, Soto V, Ortiz-Vega KM, Zarco-Márquez G, Molina-Jijón E, Cristóbal-García M, et al. Curcumin induces Nrf2 nuclear translocation and prevents glomerular hypertension, hyperfiltration, oxidant stress, and the decrease in antioxidant enzymes in 5/6 nephrectomized rats. Oxid Med Cell Longev. 2012;2012:269039. doi: 10.1155/2012/269039 22919438 PMC3424005

[55] Edelstein CL. Biomarkers of kidney disease. Second ed. Amsterdam; Boston: Elsevier/Academic Press. 2017.

[56] Landry GM, Martin S, McMartin KE. Diglycolic acid is the nephrotoxic metabolite in diethylene glycol poisoning inducing necrosis in human proximal tubule cells in vitro. Toxicol Sci. 2011;124(1):35–44. doi: 10.1093/toxsci/kfr204 21856646

[57] Abdel-Daim MM, Kilany OE, Khalifa HA, Ahmed AAM. Allicin ameliorates doxorubicin-induced cardiotoxicity in rats via suppression of oxidative stress, inflammation and apoptosis. Cancer Chemother Pharmacol. 2017;80(4):745–53. doi: 10.1007/s00280-017-3413-7 28785995

[58] Parekattil SJ, Esteves S, Agarwal A. Male infertility: contemporary clinical approaches, andrology, art and antioxidants. 2nd ed. Cham: Springer International Publishing. 2020. doi: 10.1007/978-3-030-32300-4

[59] Depeint F, Bruce WR, Shangari N, Mehta R, O’Brien PJ. Mitochondrial function and toxicity: role of the B vitamin family on mitochondrial energy metabolism. Chem Biol Interact. 2006;163(1–2):94–112. doi: 10.1016/j.cbi.2006.04.014 16765926

[60] Knight J, Jiang J, Assimos DG, Holmes RP. Hydroxyproline ingestion and urinary oxalate and glycolate excretion. Kidney Int. 2006;70(11):1929–34. doi: 10.1038/sj.ki.5001906 17021603 PMC2268952

[61] Brent J, McMartin K, Phillips S, Burkhart KK, Donovan JW, Wells M. Fomepizole for the Treatment of Ethylene Glycol Poisoning. N Engl J Med. 1999;340:832–8. doi: 10.1056/NEJM199903183401102 10080845

[62] O’Brien KL. Epidemic of pediatric deaths from acute renal failure caused by diethylene glycol poisoning. JAMA. 1998;279:1175. doi: 10.1001/jama.279.15.1175 9555756

[63] World Health Organization. Global surveillance and monitoring system for substandard and falsified medical products: activity report, August 2017-December 2021. 2024. https://www.who.int/publications/i/item/9789240097513

[64] Barceloux DG, Krenzelok EP, Olson K, Watson W. American Academy of Clinical Toxicology Practice Guidelines on the Treatment of Ethylene Glycol Poisoning. Ad Hoc Committee. J Toxicol Clin Toxicol. 1999;37(5):537–60. doi: 10.1081/clt-100102445 10497633

[65] Attaran A, Barry D, Basheer S, Bate R, Benton D, Chauvin J, et al. How to achieve international action on falsified and substandard medicines. BMJ. 2012;345:e7381. doi: 10.1136/bmj.e7381 23149211

[66] Zakhari S. Overview: how is alcohol metabolized by the body? Alcohol Res Health. 2006;29(4):245–54. 17718403 PMC6527027

