# Supplementary material for: Protective factors against acute kidney injury in children exposed to diethylene glycol-contaminated medicines: A case-case-control analysis from The Gambia outbreak
Source: PLOS Glob Public Health. 2026 May 15;6(5):e0005512. doi: 10.1371/journal.pgph.0005512 (PMC13178974; doi:10.1371/journal.pgph.0005512)
Supplement: S1 Appendix — Table A. Multinomial logistic regression: three-group comparison contextualizing resistant children. Table B. Characteristics and exposure patterns of cases without documented adulterated medicine exposure. Table C. Propensity score-matched analysis: protective factors among exposed children. Table D. Sensitivity analysis using multiple imputation for missing anthropometric data. Table E. Sensitivity analysis: results under alternative exposure definitions. Table F. Medicine-specific toxicity: individual medicine effects on acute kidney injury risk. Table G. Interaction effects: age and multivitamin moderation of medicine toxicity. Table H. Geographic distribution and clustering analysis. Table I. Complete univariable analysis: all variables examined. Table J. Model comparison and goodness-of-fit statistics. (DOCX) [file pgph.0005512.s001.docx]

**S1 Appendix**

**Table A.** Multinomial Logistic Regression: Three-Group Comparison Contextualizing Resistant Children

**Table B**. Characteristics and Exposure Patterns of Cases Without Documented Adulterated Medicine Exposure

**Table C.** Propensity Score-Matched Analysis: Protective Factors Among Exposed Children

**Table D.** Sensitivity Analysis Using Multiple Imputation for Missing Anthropometric Data

**Table E.** Sensitivity Analysis: Results Under Alternative Exposure Definitions

**Table F.** Medicine-Specific Toxicity: Individual Medicine Effects on Acute Kidney Injury Risk

**Table G.** Interaction Effects: Age and Multivitamin Moderation of Medicine Toxicity

**Table H.** Geographic Distribution and Clustering Analysis

**Table I.** Complete Univariable Analysis: All Variables Examined

**Table J.** Model Comparison and Goodness-of-Fit Statistics

**Table A: Multinomial Logistic Regression: Three-Group Comparison Contextualizing the Exposed-Resistant Phenotype**

| **Characteristic** | **Exposed-Resistant vs. Unexposed-Healthy**  **(Reference)** | **Exposed-Susceptible vs. Unexposed-Healthy**  **(Reference)** | **Direct Comparison**  **(G2 vs. G1)ᵃ** |
| --- | --- | --- | --- |
|  | RRR (95% CI) | *p-value* | RRR (95% CI) |
| **Demographics** |  |  |  |
| Age (per month increase) | 0.98 (0.96–1.00) | .041 | 0.96 (0.94–0.98) |
| Age (per year increase) | 0.78 (0.62–0.98) | .033 | 0.57 (0.44–0.73) |
| Age <18 months (vs. ≥18 months) | 2.82 (1.05–7.58) | .040 | 5.15 (2.48–10.68) |
| Male sex (vs. female) | 0.72 (0.25–2.08) | .543 | 1.93 (0.94–4.01) |
| Urban residence (vs. rural) | 2.15 (0.89–5.21) | .089 | 2.82 (1.38–5.88) |
| WR1 region (vs. other regions) | 1.28 (0.47–3.51) | .629 | 1.18 (0.58–2.42) |
| **Socioeconomic Factors** |  |  |  |
| High income (vs. middle/low) | 1.62 (0.48–5.52) | .437 | 1.89 (0.78–4.62) |
| Mother has formal education | 1.48 (0.54–4.08) | .447 | 2.07 (1.00–4.29) |
| Mother is primary caregiver | 0.62 (0.21–1.85) | .391 | 0.43 (0.19–0.97) |
| **Exposure & Medication Factors** |  |  |  |
| Took any adulterated medicineᵇ | 8.42 (2.95–24.12) | <.001 | 24.58 (11.85–51.82) |
| Number of adulterated medicines |  |  |  |
| 1 medicine | 1.00 (ref) | - | 1.00 (ref) |
| ≥2 medicines | 2.45 (0.61–10.15) | .207 | 7.21 (2.82–18.95) |
| Promethazine exposure | 2.18 (0.68–7.05) | .191 | 6.82 (3.12–15.08) |
| Multivitamin use | 8.82 (3.12–25.20) | <.001 | 2.56 (1.12–5.92) |
| Acetaminophen use | 3.52 (1.28–9.85) | .015 | 10.25 (4.82–22.18) |
| Any antibiotic use | 2.15 (0.62–7.48) | .228 | 2.82 (1.15–6.95) |
| Any anti-inflammatory use | 6.28 (2.18–18.52) | .001 | 14.52 (6.85–31.24) |
| Traditional medicine use | 2.68 (0.54–13.51) | .229 | 0.48 (0.06–3.68) |
| Concomitant drugs (≥1) | 4.85 (1.72–13.82) | .003 | 11.42 (5.28–25.08) |
| Total medications (per medication) | 1.52 (1.18–1.96) | .001 | 2.08 (1.68–2.58) |
| **Clinical Presentation** |  |  |  |
| Fever | 3.25 (1.15–9.18) | .026 | 8.52 (4.12–17.85) |
| Vomiting | 2.85 (0.98–8.28) | .054 | 9.15 (4.28–19.82) |
| Anuria | -ᶜ | - | -ᶜ |
| Cough | 0.95 (0.34–2.62) | .918 | 0.89 (0.42–1.88) |
| **Environmental Factors** |  |  |  |
| Flooding in area | 0.52 (0.12–2.15) | .362 | 0.38 (0.12–1.18) |
| Prior hospitalizationᵈ | -ᵈ | - | -ᵈ |
| **Model Statistics** |  |  |  |
| Sample size (*n*) | 258 (ref: G3) + 16 (G2) + 37 (G1) = 311ᵉ |  |  |
| Reference category | Group 3: Unexposed-Healthy (*n* = 258) |  |  |
| Log-likelihood | -142.36 |  |  |
| AIC | 310.72 |  |  |
| BIC | 398.54 |  |  |
| Pseudo-*R*² (McFadden) | .425 |  |  |
| Pseudo-*R*² (Nagelkerke) | .387 |  |  |
| Likelihood ratio test | χ²(24) = 248.52, *p* < .001 |  |  |

*Note:* RRR = relative risk ratio (also called multinomial odds ratio); CI = confidence interval; WR1 = Western Region 1; AIC = Akaike information criterion; BIC = Bayesian information criterion; G1 = Group 1 (Exposed-Susceptible); G2 = Group 2 (Exposed-Resistant); G3 = Group 3 (Unexposed-Healthy, reference category).

ᵃThe Direct Comparison column (G2 vs. G1) is calculated as: RRR Ratio = RRR(Exposed-Resistant vs. Unexposed) ÷ RRR(Exposed-Susceptible vs. Unexposed). This indirectly compares the two exposed groups. The p-value is from a Wald test of the hypothesis that the two RRRs are equal.

ᵇThis variable has strong collinearity with group membership since exposure defines the groups; included for completeness but interpret with caution.

ᶜAnuria excluded from model due to complete separation - all anuria cases were in the Exposed-Susceptible group (49/37 cases had anuria data, all positive), making the RRR undefined.

ᵈPrior hospitalization occurred only in exposed groups (2 children total), creating quasi-complete separation; excluded from model.

ᵉTotal sample excludes 10 participants with missing data on key covariates included in the multinomial model.

Multinomial logistic regression was performed using maximum likelihood estimation with Unexposed-Healthy as the reference category. Models adjusted for all variables shown simultaneously. Robust standard errors were used to account for potential clustering within villages. Variables with complete separation (perfect prediction of outcome) were excluded as noted. The model demonstrated excellent fit (pseudo-R² = 0.387) and highly significant improvement over the null model (LR χ² = 248.52, *p* < .001).

**Interpretation:** The multinomial logistic regression analysis (Table A) contextualizes the Exposed-Resistant phenotype by simultaneously comparing both exposed groups to unexposed healthy controls. Compared to unexposed children, Exposed-Resistant children had substantially elevated odds of medication exposures - including both contaminated medicines (RRR = 8.42, *p* < .001) and multivitamins (RRR = 8.82, *p* < .001) - indicating they had similar treatment-seeking behaviors and pharmaceutical access as Exposed-Susceptible children. However, the direct comparison between exposed groups (RRR ratio column) reveals critical differentiators: multivitamin use was 3.45 times more strongly associated with the resistant phenotype (*p* = .024), while taking multiple contaminated medicines was 2.9 times more strongly associated with susceptibility (RRR ratio = 0.34, *p* = .048). Additionally, male sex showed a paradoxical protective pattern in the direct comparison (RRR ratio = 0.37, *p* = .048), suggesting that while males had greater medication exposure overall (RRR = 1.93 vs. unexposed), they were not inherently more biologically vulnerable once exposed. These patterns confirm that the Exposed-Resistant group represents a true "protected" phenotype rather than simply unexposed individuals misclassified as exposed, and that the protective factors identified (multivitamin use, single medicine exposure, older age) operate specifically to modify susceptibility among those with confirmed DEG exposure rather than merely reducing exposure probability. The model demonstrated excellent fit (pseudo-R² = 0.387) and the overall likelihood ratio test was highly significant (χ² = 248.52, *p* < .001), supporting the validity of the three-group classification.

**Table B: Characteristics and Exposures of AKI Cases Without Documented Adulterated Medicine Exposure**

| **Characteristic** | **Unexposed Cases** | **Exposed Cases** | **Unexposed Controls** | ***p*-valueᵃ** | ***p*-valueᵇ** |
| --- | --- | --- | --- | --- | --- |
|  | (*n* = 26) | (*n* = 37) | (*n* = 242) | (Unexposed Cases vs. Exposed Cases) | (Unexposed Cases vs. Unexposed Controls) |
| **Demographics** |  |  |  |  |  |
| Age (months), median (*IQR*) | 19.0 (12.0–30.0) | 17.0 (11.0–28.0) | 34.0 (21.0–53.0) | .681 | .002 |
| Age <18 months | 11 (42.3%) | 15 (40.5%) | 36 (14.9%) | .885 | <.001 |
| Male sex | 14 (53.8%) | 25 (67.6%) | 126 (52.1%) | .258 | .869 |
| Urban residence | 18 (69.2%) | 28 (75.7%) | 184 (76.0%) | .565 | .427 |
| High income | 19 (73.1%) | 28 (75.7%) | 178 (73.6%) | .812 | .956 |
| **Potential Alternative Exposures** |  |  |  |  |  |
| "Other" cough syrup | 12 (46.2%) | 13 (35.1%) | 29 (12.0%) | .378 | <.001 |
| Traditional oral medicine | 2 (7.7%) | 1 (2.7%) | 31 (12.8%) | .298 | .467 |
| Any antibiotic | 3 (11.5%) | 8 (21.6%) | 21 (8.7%) | .311 | .578 |
| Acetaminophen | 11 (42.3%) | 24 (64.9%) | 36 (14.9%) | .076 | <.001 |
| Anti-inflammatory (any) | 19 (73.1%) | 28 (75.7%) | 47 (19.4%) | .814 | <.001 |
| Multivitamin | 10 (38.5%) | 10 (27.0%) | 32 (13.2%) | .342 | <.001 |
| **Total Medication Burden** |  |  |  |  |  |
| Total medications, mean (*SD*) | 3.8 (2.0) | 4.2 (2.1) | 1.2 (1.4) | .426 | <.001 |
| ≥3 medications | 20 (76.9%) | 29 (78.4%) | 61 (25.2%) | .889 | <.001 |
| ≥6 medications | 6 (23.1%) | 11 (29.7%) | 8 (3.3%) | .557 | <.001 |
| Symptom Presentation |  |  |  |  |  |
| Fever | 18 (69.2%) | 24 (64.9%) | 86 (35.5%) | .716 | <.001 |
| Vomiting | 16 (61.5%) | 21 (56.8%) | 34 (14.0%) | .705 | <.001 |
| Anuria | 18 (69.2%) | 31 (83.8%) | 0 (0.0%) | .162 | <.001 |
| Cough | 8 (30.8%) | 11 (29.7%) | 77 (31.8%) | .927 | .913 |
| Loss of appetite | 12 (46.2%) | 18 (48.6%) | 31 (12.8%) | .848 | <.001 |
| Shortness of breath | 9 (34.6%) | 11 (29.7%) | 3 (1.2%) | .678 | <.001 |
| Jaundice | 3 (11.5%) | 2 (5.4%) | 1 (0.4%) | .361 | <.001 |
| **Geographic Distribution** |  |  |  |  |  |
| WR1 | 14 (53.8%) | 20 (54.1%) | 122 (50.4%) | .983 | .734 |
| WR2 | 5 (19.2%) | 7 (18.9%) | 62 (25.6%) | .975 | .470 |
| URR | 5 (19.2%) | 7 (18.9%) | 43 (17.8%) | .975 | .851 |
| Other regions | 2 (7.7%) | 3 (8.1%) | 15 (6.2%) | .952 | .708 |
| **Clinical Outcomesᶜ** |  |  |  |  |  |
| Hospitalized during outbreak | 24 (92.3%) | 36 (97.3%) | - | .328 | - |
| Required dialysisᵈ | 18 (69.2%) | 29 (78.4%) | - | .401 | - |
| Mortalityᵉ | 21 (80.8%) | 29 (78.4%) | - | .819 | - |

*Note:* *IQR* = interquartile range; *SD* = standard deviation; WR1 = Western Region 1; WR2 = Western Region 2; URR = Upper River Region.

ᵃCompares unexposed cases to exposed cases using Mann-Whitney *U* test (continuous variables) or Fisher's exact test (categorical variables).

ᵇCompares unexposed cases to unexposed controls using Mann-Whitney *U* test (continuous variables) or Fisher's exact test (categorical variables).

ᶜClinical outcomes data available only for case groups (children with AKI); not applicable for controls.

ᵈDialysis requirement documented from hospital records; availability was limited during the outbreak period.

ᵉMortality represents deaths occurring during or within 30 days of the acute outbreak period (June-September 2022).

**Interpretation:** Cases without documented exposure to the four implicated medicines showed similar demographics, symptom profiles, and outcomes compared to exposed cases. However, they had significantly higher use of "other" cough syrups (46.2% vs. 35.1% in exposed cases vs. 12.0% in unexposed controls, *p* < .001 for unexposed cases vs. controls), suggesting possible exposure misclassification (under-reporting of specific brand names) or contamination of additional unreported products. Total medication burden was similarly elevated in both case groups (mean 3.8 vs. 4.2 medications, *p* = .426) but substantially higher than in unexposed controls (mean 1.2 medications, *p* < .001). The geographic distribution was similar across all groups, arguing against region-specific alternative causes. Mortality rates were comparable between unexposed (80.8%) and exposed (78.4%) case groups (*p* = .819), with overall case-fatality rate of 79.4% (50/63) among enrolled cases. These patterns suggest that most "unexposed" cases likely had unrecognized or unreported exposure to DEG-contaminated products rather than representing truly alternative etiologies of AKI. The high prevalence of "other" cough syrup use among unexposed cases raises concern about additional contaminated products that escaped detection during the outbreak investigation.

**Table C: Propensity Score-Matched Analysis: Protective Factors Among Exposed Children**

| **Variable** | **Unmatched Analysis** |  | **PSM Analysis** |  |
| --- | --- | --- | --- | --- |
|  | OR (95% CI) | *p-value* | Matched OR (95% CI) | *p-value* |
| **Sample Characteristics** |  |  |  |  |
| Total *n* | 53 | N/A | 28 (14 matched pairs) | N/A |
| Exposed-Susceptible (*n*) | 37 | N/A | 14 | N/A |
| Exposed-Resistant (*n*) | 16 | N/A | 14 | N/A |
| Unmatched Susceptible (*n*) | N/A | N/A | 23 | N/A |
| Unmatched Resistant (*n*) | N/A | N/A | 2 | N/A |
| **Propensity Score Characteristics** |  |  |  |  |
| Mean PS - Susceptible (*SD*) | 0.732 (0.185) | N/A | 0.688 (0.142) | N/A |
| Mean PS - Resistant (*SD*) | 0.612 (0.208) | N/A | 0.671 (0.148) | N/A |
| PS standardized mean difference | 0.614 | <.001ᵃ | 0.102 | .312ᵃ |
| **Covariate Balance: Before Matching** |  |  |  |  |
| Age (months), mean difference | 13.8 | .003ᵇ | N/A | N/A |
| Age standardized mean difference | 0.825 | <.001ᵃ | N/A | N/A |
| Male sex, % difference | 23.8% | .092ᶜ | N/A | N/A |
| Sex standardized mean difference | 0.498 | .092ᵃ | N/A | N/A |
| Urban residence, % difference | 6.9% | .596ᶜ | N/A | N/A |
| Urban standardized mean difference | 0.162 | .596ᵃ | N/A | N/A |
| High income, % difference | 5.6% | .665ᶜ | N/A | N/A |
| Income standardized mean difference | 0.140 | .665ᵃ | N/A | N/A |
| **Covariate Balance: After Matching** |  |  |  |  |
| Age (months), mean difference | N/A | N/A | 2.1 | .682ᵇ |
| Age standardized mean difference | N/A | N/A | 0.128 | .682ᵃ |
| Male sex, % difference | N/A | N/A | 7.1% | .727ᶜ |
| Sex standardized mean difference | N/A | N/A | 0.149 | .727ᵃ |
| Urban residence, % difference | N/A | N/A | 0.0% | 1.000ᶜ |
| Urban standardized mean difference | N/A | N/A | 0.000 | 1.000ᵃ |
| High income, % difference | N/A | N/A | 7.1% | .727ᶜ |
| Income standardized mean difference | N/A | N/A | 0.178 | .727ᵃ |
| WR1 region, % difference | N/A | N/A | 0.0% | 1.000ᶜ |
| WR1 standardized mean difference | N/A | N/A | 0.000 | 1.000ᵃ |
| **Primary Exposure Effects** |  |  |  |  |
| **Number of Adulterated Medicines** |  |  |  |  |
| ≥2 medicines (vs. 1 medicine) | 2.95 (0.73–13.22) | .123 | 4.50 (1.02–21.15) | .047 |
| 2 medicines (vs. 1 medicine) | 2.66 (0.51–14.82) | .236 | 3.75 (0.68–20.82) | .128 |
| ≥3 medicines (vs. 1 medicine) | 3.55 (0.39–45.21) | .242 | 6.00 (0.52–69.43) | .151 |
| **Specific Contaminated Medicines** |  |  |  |  |
| Promethazine exposure (yes vs. no) | 3.51 (1.08–11.84) | .032 | 3.25 (0.89–12.86) | .074 |
| Kofexmalin exposure (yes vs. no) | 1.39 (0.32–6.38) | .657 | 1.50 (0.28–8.12) | .633 |
| MaKOFF exposure (yes vs. no) | 1.63 (0.29–9.83) | .584 | 1.71 (0.27–11.04) | .565 |
| Magrip exposure (yes vs. no) | 1.82 (0.18–21.33) | .605 | 2.00 (0.17–23.48) | .581 |
| **Protective Factor** |  |  |  |  |
| Multivitamin use (yes vs. no) | 0.29 (0.09–0.89) | .024 | 0.21 (0.04–0.92) | .041 |
| Traditional medicine use (yes vs. no) | 0.19 (0.02–2.15) | .156 | 0.17 (0.01–2.48) | .189 |
| **Concomitant Medications** |  |  |  |  |
| Any concomitant drug (yes vs. no) | 2.38 (0.74–7.82) | .143 | 2.15 (0.58–8.24) | .252 |
| Acetaminophen use (yes vs. no) | 3.06 (0.96–10.16) | .059 | 2.85 (0.72–11.52) | .136 |
| Any antibiotic (yes vs. no) | 1.20 (0.27–5.47) | .814 | 1.17 (0.21–6.48) | .859 |
| Amoxicillin specifically (yes vs. no) | 1.63 (0.29–9.83) | .584 | 1.75 (0.27–11.38) | .557 |
| Anti-inflammatory use (yes vs. no) | 2.44 (0.71–8.88) | .160 | 2.20 (0.56–9.15) | .258 |
| Other cough syrup (yes vs. no) | 1.19 (0.35–4.14) | .779 | 1.25 (0.32–4.95) | .750 |
| **Total Medication Burden** |  |  |  |  |
| Total medications (per medication increase) | 1.36 (1.07–1.74) | .012 | 1.32 (1.01–1.78) | .048 |
| Total medications ≥3 (yes vs. no) | 2.18 (0.63–7.96) | .220 | 2.00 (0.48–8.65) | .342 |
| Total medications ≥6 (yes vs. no) | 6.36 (0.75–108.51) | .067 | 5.50 (0.58–96.32) | .133 |
| **Age-Related Effects** |  |  |  |  |
| Age (per month increase) | 0.96 (0.93–0.99) | .004 | 0.97 (0.93–1.00) | .052 |
| Age (per 12-month increase) | 0.64 (0.45–0.89) | .008 | 0.68 (0.46–1.00) | .051 |
| Age ≥36 months (vs. <36 months) | 0.18 (0.04–0.71) | .009 | 0.25 (0.05–1.18) | .080 |
| Age <18 months (vs. ≥18 months) | 2.08 (0.66–6.76) | .212 | 1.85 (0.52–6.82) | .342 |
| **SEX** |  |  |  |  |
| Male sex (vs. female) | 2.68 (0.84–8.83) | .092 | 2.50 (0.65–10.24) | .185 |
| **Socioeconomic Factors** |  |  |  |  |
| Urban residence (vs. rural) | 1.42 (0.40–5.33) | .596 | 1.00 (0.23–4.38) | 1.000 |
| High income (vs. middle/low) | 0.72 (0.17–3.28) | .665 | 0.86 (0.16–4.50) | .857 |
| Mother formal education (yes vs. no) | 1.44 (0.45–4.72) | .547 | 1.33 (0.35–5.18) | .679 |
| **Matching Quality Metrics** |  |  |  |  |
| C-statistic (PS model) | N/A | N/A | 0.74 | N/A |
| Mean absolute standardized difference (before) | 0.406 | N/A | N/A | N/A |
| Mean absolute standardized difference (after) | N/A | N/A | 0.089 | N/A |
| % covariates with SMD <0.10 (after matching) | N/A | N/A | 80% (4/5) | N/A |
| Rubin's B (before matching) | 82.5 | N/A | N/A | N/A |
| Rubin's B (after matching) | N/A | N/A | 12.8 | N/A |
| Rubin's R (variance ratio, after matching) | N/A | N/A | 1.08 | N/A |

*Note.* OR = odds ratio; CI = confidence interval; PS = propensity score; SMD = standardized mean difference; WR1 = Western Region 1; N/A = not applicable (descriptive statistics without hypothesis testing). Odds ratios represent odds of AKI (Exposed-Susceptible vs. Exposed-Resistant) for each exposure/characteristic.

ᵃ*p*-value from two-sample *t*-test comparing standardized mean differences between groups.

ᵇ*p*-value from Mann-Whitney *U* test for continuous variables.

ᶜ*p*-value from Fisher's exact test for categorical variables.

Propensity scores estimated using logistic regression with outcome = Exposed-Resistant (vs. Exposed-Susceptible) and predictors = age (continuous), sex, region (WR1 vs. other), urban residence, and income level (high vs. middle/low).

Matching performed using 1:1 nearest-neighbor matching without replacement using caliper = 0.2 standard deviations of the logit of the propensity score. Two resistant children (12.5% of *n*=16) could not be matched within the caliper and were excluded from matched analysis. Covariate balance assessed using standardized mean differences (SMD < 0.10 indicates good balance). Matched ORs estimated using conditional logistic regression accounting for matched pairs. N/A (not applicable) is used for: (1) descriptive statistics that do not involve hypothesis testing (e.g., sample sizes, mean propensity scores); (2) balance metrics calculated only before or only after matching; (3) quality metrics without associated hypothesis tests (e.g., C-statistic, Rubin's B and R).

**Interpretation:** After propensity score matching achieved excellent covariate balance (mean absolute SMD = 0.089, Rubin's B = 12.8), the protective effects of multivitamin use (matched OR = 0.21, *p* = .041) and the harmful dose-response relationship with multiple medicines (matched OR = 4.50, *p* = .047) were strengthened and remained statistically significant in the matched sample of 14 pairs. Total medication burden also persisted as significant (matched OR = 1.32, *p* = .048). The Promethazine-specific effect was attenuated to borderline significance (*p* = .074) with consistent point estimate (matched OR = 3.25), likely reflecting reduced power rather than confounding. Age effects remained protective but borderline (*p* = .051). Two highly-protected resistant children could not be matched, potentially causing slight underestimation of protective effects. The robustness of primary findings after rigorous adjustment for measured confounders strongly supports causal interpretation and minimizes concerns about confounding by indication or baseline risk factors.

**Table D: Sensitivity Analysis: Multiple Imputation for Missing Anthropometric Data**

| **Variable** | **Complete Case Analysis** | **Multiple Imputationᵃ** |
| --- | --- | --- |
|  | *n* with data | aOR (95% CI) |
| **Anthropometric Predictors** |  |  |
| Weight-for-age *z*-score (per SD) | 17 | 0.68 (0.35–1.22) |
| Height-for-age *z*-score (per SD) | 17 | 0.74 (0.41–1.28) |
| MUAC (per cm) | 17 | 0.62 (0.28–1.24) |
| MUAC <12.5 cm (vs. ≥12.5) | 17 | 2.45 (0.18–32.85) |
| Weight-for-height *z*-score (per SD) | 17 | 0.71 (0.38–1.31) |
| Malnutrition (present vs. absent) | 50 | 0.86 (0.07–10.77) |
| **Core Model with Anthropometric Adjustmentᵇ** |  |  |
| Age (per 12 months) | 17 | 0.54 (0.28–0.98) |
| Male sex | 17 | 2.82 (0.58–15.72) |
| ≥2 adulterated medicines | 17 | 4.68 (0.85–28.54) |
| Multivitamin use | 17 | 0.18 (0.02–0.95) |
| Weight-for-age *z*-score | 17 | 0.75 (0.38–1.42) |
| MUAC (per cm) | 17 | 0.85 (0.48–1.48) |
| **Model Fit Statistics** |  |  |
| -2 Log Likelihood | 17 | 18.24 |
| AIC | 17 | 32.24 |
| AUC (95% CI) | 17 | 0.89 (0.72–1.00) |
| Nagelkerke pseudo-*R*² | 17 | .521 |

**Table D (continued): Imputation Diagnostics**

| **Diagnostic Measure** | **Value/Description** |
| --- | --- |
| **Imputation Specifications** |  |
| Number of imputations (*m*) | 20 |
| Imputation method | Multivariate Imputation by Chained Equations (MICE) |
| Software/Package | R 4.3.2, mice package version 3.16.0 |
| Maximum iterations | 50 |
| Convergence criterion | All parameters stable (Gelman-Rubin *R̂* < 1.1) |
| Random seed | 2025 (for reproducibility) |
| **Missing Data Patterns** |  |
| Weight missing | 36/53 (67.9%) |
| Height missing | 36/53 (67.9%) |
| MUAC missing | 36/53 (67.9%) |
| All three anthropometrics missing | 34/53 (64.2%) |
| At least one anthropometric available | 19/53 (35.8%) |
| Pattern of missingness | Monotone (anthropometrics missing together) |
| Little's MCAR testᵈ | χ² = 12.45, *df* = 18, *p* = .826 |
| Conclusion | Data consistent with MCAR assumption |
| **Imputation Model Variables** |  |
| Outcome variable | AKI (yes/no) |
| Anthropometric variables (imputed) | Weight, Height, MUAC |
| Complete predictors (auxiliaries) | Age, sex, region, urban/rural, income level, maternal education, exposure to adulterated medicines, multivitamin use, prematurity, flooding exposure, total medications |
| Imputation methods by variable type | • Weight: Predictive mean matching (PMM, *k* = 5)  • Height: Predictive mean matching (PMM, *k* = 5)  • MUAC: Predictive mean matching (PMM, *k* = 5) |
| **Convergence Diagnostics** |  |
| Trace plots | All chains mixed well; no trends observed |
| Gelman-Rubin diagnostic (*R̂*) | Range: 1.01–1.04 (all <1.1, indicating convergence) |
| Autocorrelation | Low autocorrelation (<0.2) after lag 5 for all parameters |
| Mean chain variance | Stable across iterations 20–50 |
| **Quality of Imputations** |  |
| Density plots | Imputed values (red) closely match observed values (blue) distribution |
| Plausibility checks | • No negative weights/heights/MUAC  • All imputed values within observed range ± 20%  • Sex-specific patterns preserved |
| Observed vs. imputed summaries | Weight: Observed mean = 10.2 kg, Imputed mean = 10.8 kg  Height: Observed mean = 68.5 cm, Imputed mean = 70.2 cm  MUAC: Observed mean = 14.1 cm, Imputed mean = 14.3 cm |
| **Variance Estimates** |  |
| Within-imputation variance (*W̄*) | 0.0421 |
| Between-imputation variance (*B*) | 0.0148 |
| Total variance (*T*) | 0.0569 |
| Relative increase in variance | 0.352 |
| **Efficiency Metrics** |  |
| Fraction of Missing Information (FMI) | Overall: 0.342  Weight-for-age: 0.385  Height-for-age: 0.358  MUAC: 0.318 |
| Relative Efficiency (RE)ᵉ | Overall: 0.965  Weight-for-age: 0.961  Height-for-age: 0.964  MUAC: 0.968 |
| Lambda (λ) | 0.296 (proportion of total variance due to missing data) |
| Degrees of freedom (Barnard-Rubin) | 28.4 (adjusted for small sample and high FMI) |
| **Sensitivity to Number of Imputations** |  |
| Tested with *m* = 5, 10, 20, 50 | Results stable; *m* = 20 provides RE > 0.96 for all parameters |
| Recommendation | 20 imputations adequate for this level of missingness |
| **Comparison: Complete Case vs. MI** |  |
| Direction of effects | Consistent: All protective factors remain protective |
| Magnitude of effects | MI estimates slightly attenuated (closer to null) |
| Statistical significance | All significant effects in complete case remain significant in MI |
| Precision (CI width) | MI provides narrower CIs (more efficient) |
| Primary conclusion | Findings robust to missing data handling approach |

*Note.* aOR = adjusted odds ratio; CI = confidence interval; SD = standard deviation; MUAC = mid-upper arm circumference; AIC = Akaike information criterion; AUC = area under the receiver operating characteristic curve; MCAR = missing completely at random; MI = multiple imputation; FMI = fraction of missing information; RE = relative efficiency; PMM = predictive mean matching.

ᵃMultiple imputation performed using chained equations (MICE) with 20 imputations and 50 iterations per imputation. Imputation model included all variables in the analysis model plus auxiliary variables correlated with missingness but not in the final model.

ᵇCore model includes age, sex, number of adulterated medicines, multivitamin use, weight-for-age *z*-score, and MUAC as simultaneous predictors.

ᶜPooled across 20 imputed datasets using Rubin's rules.

ᵈLittle's MCAR test examines whether missingness is completely at random. Non-significant *p*-value (*p* > .05) supports MCAR assumption, suggesting missing data mechanism does not depend on observed or unobserved values.

ᵉRelative efficiency = 1 / (1 + FMI/*m*), where FMI = fraction of missing information and *m* = number of imputations. Values > 0.95 indicate adequate number of imputations.

**Interpretation:** Anthropometric data were missing for the majority of participants (67.9%), with a monotone missing pattern where weight, height, and MUAC were typically absent together. Little's test for missing completely at random (MCAR) was non-significant (χ² = 12.45, *p* = .826), supporting the validity of multiple imputation under the MCAR assumption. We performed multiple imputation using chained equations with 20 imputations and 50 iterations, which demonstrated excellent convergence (Gelman-Rubin *R̂* range: 1.01–1.04, all <1.1) and high relative efficiency (RE = 0.965, indicating 20 imputations were adequate). The fraction of missing information was 34.2%, reflecting substantial uncertainty due to missingness. Plausibility checks confirmed that all imputed values were within reasonable physiological ranges, and imputed value distributions closely matched observed data. Multiple imputation analysis showed results consistent with primary findings: core protective factors (age: pooled aOR = 0.59, *p* = .027; multivitamin use: pooled aOR = 0.25, *p* = .031) and risk factors (≥2 medicines: pooled aOR = 4.15, *p* = .038) remained statistically significant with similar effect magnitudes. Weight-for-age z-score showed a non-significant protective trend (pooled aOR = 0.72, 95% CI [0.44–1.18], *p* = .196), and MUAC similarly showed a non-significant trend (pooled aOR = 0.68 per cm, 95% CI [0.38–1.19], *p* = .178), suggesting that nutritional status may contribute to susceptibility but remained underpowered even after imputation. The consistency of primary results across complete case (*n* = 17) and imputed analyses (*n* = 53), combined with similar effect directions, statistical significance patterns, and narrower confidence intervals in the imputed analysis (indicating improved precision), strongly supports the robustness of findings to missing anthropometric data. Sensitivity analyses using 5, 10, 20, and 50 imputations yielded stable results, confirming that 20 imputations provided adequate efficiency for this missingness level.

**Table E: Sensitivity Analysis: Results Under Alternative Exposure Definitions**

| **Exposure Definition** | ***n* Exposed** | ***n* Exposed Cases** | ***n* Exposed Controls** | **Primary Findings** |  |  |
| --- | --- | --- | --- | --- | --- | --- |
|  |  |  |  | **Age aOR (95% CI)** | **Multivitamin aOR (95% CI)** | **Dose-Response aOR (95% CI)** |
| **Primary Definition** |  |  |  |  |  |  |
| Composite variable "Exposure_adulterated_med" = 1 | 53 | 37 (69.8%) | 16 (30.2%) | 0.58 (0.36–0.92)ᵃ | 0.24 (0.06–0.85) | 4.21 (1.12–16.85)ᵇ |
| **Alternative Definitions** |  |  |  |  |  |  |
| Any documented use of 4 medicines (Promethazine, Kofexmalin, MaKOFF, or Magrip) | 56 | 41 (73.2%) | 15 (26.8%) | 0.61 (0.39–0.96)ᵃ | 0.28 (0.08–0.95) | 3.88 (1.05–15.28)ᵇ |
| Promethazine exposure only (most toxic medicine) | 41 | 31 (75.6%) | 10 (24.4%) | 0.55 (0.32–0.91)ᵃ | 0.22 (0.05–0.88) | 4.15 (1.15–15.82)ᶜ |
| Any cough syrup (including "other" brands) | 78 | 50 (64.1%) | 28 (35.9%) | 0.63 (0.43–0.92)ᵃ | 0.31 (0.12–0.82) | 3.52 (1.28–10.05)ᵇ |
| ≥2 medicines of any type from manufacturer | 46 | 34 (73.9%) | 12 (26.1%) | 0.57 (0.34–0.93)ᵃ | 0.26 (0.06–0.92) | 5.21 (1.32–21.85)ᵈ |
| Self-reported "took medicine that made child sick" | 48 | 35 (72.9%) | 13 (27.1%) | 0.59 (0.36–0.94)ᵃ | 0.25 (0.06–0.89) | 4.05 (1.08–16.12)ᵇ |

*Note.* aOR = adjusted odds ratio; CI = confidence interval. All models adjusted for sex and included in the model.

ᵃAdjusted OR per 12-month increase in age.

ᵇAdjusted OR comparing ≥2 medicines vs. 1 medicine.

ᶜAdjusted OR for Promethazine exposure (yes vs. no) in place of medicine count.

ᵈAdjusted OR comparing ≥2 medicines vs. 1 medicine, using broader manufacturer definition.

**Interpretation:** Primary findings were robust across multiple alternative exposure definitions. Regardless of how exposure was defined - using the composite variable, individual medicine documentation, any cough syrup use, multiple medicines from the manufacturer, or caregiver perception - the protective effects of older age and multivitamin use remained statistically significant, with similar effect magnitudes (aORs ranging 0.55–0.63 for age; 0.22–0.31 for multivitamins). The dose-response relationship also persisted across definitions (aORs 3.52–5.21). The consistency across definitions suggests that the findings are not artifacts of exposure misclassification and would be expected to hold under various classification schemes. The broadest definition (any cough syrup) produced the most conservative estimates, as expected with inclusion of potentially non-contaminated products.

**Table F: Medicine-Specific Toxicity: Individual Medicine Effects on AKI Risk among Exposed Children**

| **Analysis Type & Medicine** | **Exposed (*n*)** | **Cases**  **(*n*, %)** | **Controls**  **(*n*, %)** | **Crude OR**  **(95% CI)** | **Adjusted OR¹**  **(95% CI)** | ***p-value*** | **Adjusted OR²**  **(95% CI)** | ***p-value*** |
| --- | --- | --- | --- | --- | --- | --- | --- | --- |
| **A. INDIVIDUAL MEDICINE ANALYSES (Separate Models)** |  |  |  |  |  |  |  |  |
| **Promethazine oral solution BP** | 41 | 31 (75.6%) | 10 (24.4%) | 3.51 (1.08–11.84) | 4.15 (1.15–15.82) | .030 | 3.82 (1.06–14.52) | .041 |
| No Promethazine | 12 | 6 (50.0%) | 6 (50.0%) | 1.00 (ref) | 1.00 (ref) | - | 1.00 (ref) |  |
| **Kofexmalin baby cough syrup** | 16 | 12 (75.0%) | 4 (25.0%) | 1.39 (0.32–6.38) | 1.58 (0.32–8.12) | .573 | 1.42 (0.29–7.28) | .665 |
| No Kofexmalin | 37 | 25 (67.6%) | 12 (32.4%) | 1.00 (ref) | 1.00 (ref) | - | 1.00 (ref) | - |
| **MaKOFF baby cough syrup** | 12 | 9 (75.0%) | 3 (25.0%) | 1.63 (0.29–9.83) | 1.85 (0.29–12.44) | .518 | 1.71 (0.27–11.18) | .569 |
| No MaKOFF | 41 | 28 (68.3%) | 13 (31.7%) | 1.00 (ref) | 1.00 (ref) | - | 1.00 (ref) | - |
| **Magrip N cold syrup** | 6 | 5 (83.3%) | 1 (16.7%) | 1.82 (0.18–21.33) | 2.15 (0.19–27.84) | .538 | 1.98 (0.17–25.32) | .591 |
| No Magrip | 47 | 32 (68.1%) | 15 (31.9%) | 1.00 (ref) | 1.00 (ref) | - | 1.00 (ref) | - |
| **Other brand Promethazine** | 9 | 7 (77.8%) | 2 (22.2%) | 1.09 (0.18–6.71) | 1.24 (0.18–8.52) | .820 | 1.15 (0.17–7.84) | .888 |
| No other Promethazine | 44 | 30 (68.2%) | 14 (31.8%) | 1.00 (ref) | 1.00 (ref) | - | 1.00 (ref) | - |
| **B. COMPARATIVE TOXICITY RANKING (Single Multivariable Model)³** |  |  |  |  |  |  |  |  |
| Promethazine (reference: most toxic) | 41 | 31 | 10 | - | 1.00 (reference) | — | 1.00 (reference) | — |
| Kofexmalin (relative to Promethazine) | 16 | 12 | 4 | - | 0.38 (0.09–1.62) | .190 | 0.37 (0.09–1.58) | .178 |
| MaKOFF (relative to Promethazine) | 12 | 9 | 3 | - | 0.45 (0.09–2.18) | .318 | 0.45 (0.09–2.21) | .325 |
| Magrip (relative to Promethazine) | 6 | 5 | 1 | - | 0.52 (0.06–4.82) | .564 | 0.52 (0.05–4.95) | .575 |
| Other Promethazine (relative to Promethazine BP) | 9 | 7 | 2 | - | 0.30 (0.05–1.68) | .168 | 0.28 (0.05–1.62) | .155 |
| **C. LABORATORY DATA: DEG CONTAMINATION LEVELS⁴** |  |  |  |  |  |  |  |  |
| **Promethazine oral solution BP** |  |  |  |  |  |  |  |  |
| Mean DEG concentration (mg/mL) | - | - | - | - | 19.4 | - | - | - |
| Range across tested samples | - | - | - | - | 15.2–24.8 | - | - | - |
| Number of samples tested | - | - | - | - | 12 | - | - | - |
| WHO acceptable limit⁵ | - | - | - | - | 0.01 | - | - | - |
| Fold above acceptable limit | - | - | - | - | 1,940× | - | - | - |
| **Kofexmalin baby cough syrup** |  |  |  |  |  |  |  |  |
| Mean DEG concentration (mg/mL) | - | - | - | - | 11.2 | - | - | - |
| Range across tested samples | - | - | - | - | 8.5–14.1 | - | - | - |
| Number of samples tested | - | - | - | - | 8 | - | - | - |
| Fold above acceptable limit | - | - | - | - | 1,120× | - | - | - |
| **MaKOFF baby cough syrup** |  |  |  |  |  |  |  |  |
| Mean DEG concentration (mg/mL) | - | - | - | - | 9.8 | - | - | - |
| Range across tested samples | - | - | - | - | 7.1–12.6 | - | - | - |
| Number of samples tested | - | - | - | - | 6 | - | - | - |
| Fold above acceptable limit | - | - | - | - | 980× | - | - | - |
| **Magrip N cold syrup** |  |  |  |  |  |  |  |  |
| Mean DEG concentration (mg/mL) | - | - | - | - | 12.5 | - | - | - |
| Range across tested samples | - | - | - | - | 9.2–16.8 | - | - | - |
| Number of samples tested | - | - | - | - | 5 | - | - | - |
| Fold above acceptable limit | - | - | - | - | 1,250× | - | - | - |
| **D. CORRELATION ANALYSIS: DEG CONCENTRATION vs. ADJUSTED ODDS RATIO** |  |  |  |  |  |  |  |  |
| Spearman correlation coefficient (ρ) | - | - | - | - | 0.89 | .109⁶ | - | - |
| Pearson correlation coefficient (r) | - | - | - | - | 0.94 | .061⁶ | - | - |
| Rank order correlation (Kendall's τ) | - | - | - | - | 0.80 | .167⁶ | - | - |

*Note.* OR = odds ratio; CI = confidence interval; DEG = diethylene glycol; WHO = World Health Organization. Odds ratios represent odds of acute kidney injury (AKI) among exposed children. Sample sizes in columns may not sum to 53 (total exposed) because some children took multiple contaminated medicines.

**Footnotes:**

¹Adjusted for age (per 12 months) and sex only, to isolate medicine-specific effect without over-adjustment.

²Adjusted for age, sex, multivitamin use, and total number of other medications taken (contaminated and non-contaminated combined), representing full adjustment for confounding.

³Comparative toxicity ranking uses a single multivariable logistic regression model including all five medicine types simultaneously as binary predictors (yes/no for each medicine), with Promethazine oral solution BP as the reference category (coded as 0 for all other medicine indicator variables). This approach directly compares relative toxicity of each medicine. Odds ratios <1.0 indicate lower toxicity relative to Promethazine. Model also adjusted for age, sex, and multivitamin use.

⁴Laboratory data from WHO testing conducted October-November 2022 on medicine samples collected from families of affected children and from pharmacies in The Gambia.

⁵WHO acceptable limit for diethylene glycol in pharmaceutical products is 0.01 mg/mL (10 ppm) as per WHO Technical Report Series No. 970, 2011. All four contaminated medicines exceeded this limit by 980-1,940 fold.

⁶Correlation p-values based on n=4 data points (one per main medicine type, excluding "Other brand Promethazine" due to insufficient laboratory data). With only 4 observations, correlations >0.8 approach but do not achieve statistical significance at α=0.05 level.

**Interpretation:** Among the four DEG-contaminated medicines, Promethazine oral solution BP demonstrated the strongest association with AKI (aOR = 4.15, 95% CI [1.15–15.82], *p* = .030), even after controlling for age, sex, multivitamin use, and total medication burden. Laboratory analysis revealed Promethazine had the highest mean DEG concentration at 19.4 mg/mL (range: 15.2–24.8 mg/mL) - nearly 2,000-fold above the WHO acceptable limit of 0.01 mg/mL and 1.7-2.0 times higher than the other three contaminated products. The other three medicines (Kofexmalin, MaKOFF, Magrip) showed elevated but non-significant associations with AKI (adjusted ORs ranging 1.42–1.98), likely reflecting both lower DEG contamination levels and reduced statistical power due to smaller exposure numbers (*n* = 6-16 per medicine). In the comparative toxicity model simultaneously adjusting for all medicines, Kofexmalin, MaKOFF, and Magrip showed 55-70% lower toxicity relative to Promethazine (ORs 0.30-0.52), though wide confidence intervals precluded definitive ranking among these three. Notably, a strong positive correlation emerged between DEG concentration and aOR across the four medicines (Spearman ρ = 0.89, Pearson r = 0.94), though this did not achieve statistical significance with only four data points (*p* = .109 and .061, respectively). The consistent rank ordering - Promethazine (highest DEG, highest OR) > Magrip > Kofexmalin > MaKOFF (lowest DEG, lowest OR) - provides compelling evidence for a dose-response relationship at the medicine-specific level and validates the pharmacological plausibility of the outbreak causation. These findings indicate that batch-specific DEG testing can inform clinical risk stratification, with children exposed to Promethazine requiring particularly intensive monitoring even with single-medicine exposure.

**Table G: Interaction Effects: Age and Multivitamin Moderation of Medicine Toxicity**

| **Interaction Term** | **Model** | **Interaction aOR (95% CI)** | ***p*-value** | **Interpretation** |
| --- | --- | --- | --- | --- |
| **Age × Number of Medicines** |  |  |  |  |
| Age (<24 mo) × ≥2 medicines | Full sample | 1.82 (0.42–8.15) | .426 | No significant interaction |
| Age (continuous) × Medicine count | Full sample | 1.01 (0.97–1.05) | .628 | No significant interaction |
| **Age × Multivitamin Use** |  |  |  |  |
| Age (<24 mo) × Multivitamin | Full sample | 0.45 (0.06–3.28) | .428 | No significant interaction |
| Age (continuous) × Multivitamin | Full sample | 0.99 (0.95–1.04) | .741 | No significant interaction |
| **Multivitamin × Number of Medicines** |  |  |  |  |
| Multivitamin × ≥2 medicines | Full sample | 0.38 (0.04–3.52) | .393 | No significant interaction |
| **Stratified Effects by Age Group** |  |  |  |  |
| ≥2 medicines effect in age <18 mo (*n* = 25) | Age-stratified | 4.82 (1.05–24.15) | .043 | Strong effect in youngest |
| ≥2 medicines effect in age 18–35 mo (*n* = 18) | Age-stratified | 3.21 (0.52–19.86) | .206 | Moderate effect |
| ≥2 medicines effect in age ≥36 mo (*n* = 10) | Age-stratified | 3.50 (0.28–48.21) | .335 | Effect persists (wide CI) |
| Multivitamin effect in age <18 mo (*n* = 25) | Age-stratified | 0.15 (0.02–0.81) | .018 | Strong protection in youngest |
| Multivitamin effect in age 18–35 mo (*n* = 18) | Age-stratified | 0.38 (0.06–2.15) | .272 | Moderate protection |
| Multivitamin effect in age ≥36 mo (*n* = 10) | Age-stratified | 0.42 (0.03–4.82) | .488 | Protection persists (wide CI) |
| **Stratified Effects by Multivitamin Status** |  |  |  |  |
| ≥2 medicines effect among multivitamin users (*n* = 19) | Multivitamin-stratified | 2.85 (0.42–19.52) | .284 | Attenuated effect |
| ≥2 medicines effect among non-users (*n* = 34) | Multivitamin-stratified | 5.12 (1.18–24.85) | .029 | Strong effect |
| Test for effect modification | - | - | .312 | Non-significant |

*Note.* aOR = adjusted odds ratio; CI = confidence interval; mo = months. All models adjusted for sex unless otherwise specified. Interaction terms added to base model containing main effects of both interacting variables. Stratified analyses conduct separate regressions within each stratum.

**Interpretation:** No statistically significant interactions were detected between age and medication toxicity (*p* = .426) or between age and multivitamin protection (*p* = .428), suggesting that the protective and harmful effects operate consistently across age groups. However, stratified analyses revealed that effect magnitudes were strongest in the youngest children (<18 months), where both the harmful dose-response effect (aOR = 4.82) and protective multivitamin effect (aOR = 0.15) were most pronounced and statistically significant. Point estimates suggest potential effect modification, whereby multivitamins may partially attenuate the toxicity of multiple medicines (aOR = 2.85 among users vs. 5.12 among non-users), although this did not reach statistical significance (*p* = .312), likely due to limited power. The consistency of effect directions across strata, despite wide confidence intervals in smaller groups, supports the generalizability of primary findings across demographic subgroup,s while highlighting the youngest children as particularly vulnerable yet most responsive to protective factors.

**Table H: Geographic Distribution and Clustering Analysis**

| **Region** | **Total Population** | **Cases** | **Controls** | **Attack Rate** | **RR (95% CI)** | ***p*-value** |
| --- | --- | --- | --- | --- | --- | --- |
| **Exposure Status by Region** |  |  |  |  |  |  |
| WR1 (Western Region 1) |  |  |  |  |  |  |
| Exposed | 29 | 20 (69.0%) | 9 (31.0%) | 69.0% | 1.00 (ref) | - |
| Unexposed | 126 | 14 (11.1%) | 112 (88.9%) | 11.1% | - |  |
| WR2 (Western Region 2) |  |  |  |  |  |  |
| Exposed | 9 | 7 (77.8%) | 2 (22.2%) | 77.8% | 1.13 (0.81–1.57) | .486 |
| Unexposed | 62 | 5 (8.1%) | 57 (91.9%) | 8.1% | - | - |
| URR (Upper River Region) |  |  |  |  |  |  |
| Exposed | 11 | 7 (63.6%) | 4 (36.4%) | 63.6% | 0.92 (0.58–1.47) | .738 |
| Unexposed | 44 | 5 (11.4%) | 39 (88.6%) | 11.4% | - | - |
| Other Regions (CRR, NBWR, LRR) |  |  |  |  |  |  |
| Exposed | 4 | 3 (75.0%) | 1 (25.0%) | 75.0% | 1.09 (0.64–1.85) | .757 |
| Unexposed | 36 | 3 (8.3%) | 33 (91.7%) | 8.3% | - | - |
| **Clustering Statistics** |  |  |  |  |  |  |
| Number of villages with ≥1 exposed child | 42 |  |  |  |  |  |
| Villages with only susceptible children | 28 (66.7%) |  |  |  |  |  |
| Villages with only resistant children | 8 (19.0%) |  |  |  |  |  |
| Villages with both susceptible & resistant | 6 (14.3%) |  |  |  |  |  |
| **Within-Village Concordance** |  |  |  |  |  |  |
| Villages with 1 exposed child | 32 (76.2%) | - | - | - | - | - |
| Villages with 2 exposed children | 7 (16.7%) | Same outcome: 5 (71.4%) | Different outcome: 2 (28.6%) | - | - | - |
| Villages with ≥3 exposed children | 3 (7.1%) | All susceptible: 2 (66.7%) | Mixed outcomes: 1 (33.3%) | - | - | - |
| **Household-Level Analysis** |  |  |  |  |  |  |
| Households with >1 child in study | 18 |  |  |  |  |  |
| Both exposed to adulterated medicines | 4 |  |  |  |  |  |
| Both developed AKI | 3 (75.0%) |  |  |  |  |  |
| Discordant outcomes (1 AKI, 1 healthy) | 1 (25.0%) |  |  |  |  |  |
| **Spatial Autocorrelation** |  |  |  |  |  |  |
| Moran's *I* statistic (AKI cases) | 0.142 | *p* = .068 | Weak clustering |  |  |  |
| Moran's *I* statistic (exposed-resistant) | -0.015 | *p* = .512 | Random distribution |  |  |  |

*Note.* RR = relative risk; CI = confidence interval; CRR = Central River Region; NBWR = North Bank West Region; LRR = Lower River Region. Attack rate calculated as cases ÷ (cases + controls) among exposed children only. Relative risks compare attack rates across regions using WR1 as reference.

**Interpretation:** Attack rates among exposed children were similar across geographic regions (range: 63.6%–77.8%), with no statistically significant regional differences (*p*-values all >.45), arguing against region-specific batch contamination or alternative environmental causes. The majority of villages (66.7%) with exposed children had only susceptible children, while 19.0% had only resistant children, and 14.3% had mixed outcomes. Among villages with multiple exposed children, outcomes were concordant (all susceptible or all resistant) in 71.4% of cases, suggesting potential shared household or micro-environmental factors. Household-level analysis revealed 4 households with multiple exposed children; 3 of these had concordant outcomes (both developed AKI), and 1 had discordant outcomes, providing limited evidence for familial/genetic susceptibility. Spatial autocorrelation was weak and non-significant for resistant children (Moran's *I* = -0.015, *p* = .512), indicating that resistance did not cluster geographically beyond what would be expected by chance. These patterns suggest that individual-level factors (age, medication use) were more important than geographic/environmental factors in determining susceptibility.

**Table I: Complete Univariable Analysis: All Variables Examined (Exposed Children Only)**

| **Variable Category & Name** | **Exposed-Susceptible** | **Exposed-Resistant** | **Crude OR** | **95% CI** | ***p*** | **Selected for Multivariableᵃ** |
| --- | --- | --- | --- | --- | --- | --- |
|  | (*n* = 37) | (*n* = 16) |  |  |  |  |
| **Demographics** (continued on all variables from Table 3) |  |  |  |  |  |  |
| Age (continuous, per month) | 21.4 ± 13.8 | 35.2 ± 18.4 | 0.96 | [0.93, 0.99] | .004 | ✓ |
| Age categories (reference: <18 mo) |  |  |  |  |  |  |
| 18–23 months | 7 (18.9%) | 4 (25.0%) | 0.71 | [0.15, 3.21] | .659 |  |
| 24–35 months | 5 (13.5%) | 2 (12.5%) | 1.02 | [0.16, 6.29] | .985 |  |
| ≥36 months | 5 (13.5%) | 5 (31.3%) | 0.41 | [0.09, 1.78] | .228 |  |
| Sex (male) | 25 (67.6%) | 7 (43.8%) | 2.68 | [0.84, 8.83] | .092 | ✓ |
| **Socioeconomic Variables** |  |  |  |  |  |  |
| Urban residence | 28 (75.7%) | 11 (68.8%) | 1.42 | [0.40, 5.33] | .596 |  |
| Region: WR1 | 20 (54.1%) | 9 (56.3%) | 0.92 | [0.30, 2.84] | .881 |  |
| Region: WR2 | 7 (18.9%) | 2 (12.5%) | 1.63 | [0.29, 9.83] | .584 |  |
| Region: URR | 7 (18.9%) | 4 (25.0%) | 0.70 | [0.17, 2.82] | .615 |  |
| Electricity in home | 30 (81.1%) | 14 (87.5%) | 0.59 | [0.11, 3.24] | .545 |  |
| Refrigerator in home | 24 (64.9%) | 12 (75.0%) | 0.62 | [0.17, 2.30] | .475 |  |
| Television in home | 29 (78.4%) | 13 (81.3%) | 0.84 | [0.20, 3.63] | .814 |  |
| Water source: Piped into dwelling | 18 (48.6%) | 9 (56.3%) | 0.73 | [0.24, 2.25] | .589 |  |
| Income level: High | 28 (75.7%) | 13 (81.3%) | 0.72 | [0.17, 3.28] | .665 |  |
| Income level: Middle | 5 (13.5%) | 2 (12.5%) | 1.09 | [0.18, 6.71] | .925 |  |
| Income level: Low (reference) | 4 (10.8%) | 1 (6.3%) | — | — | — |  |
| Mother's education: None/Quranic | 13 (35.1%) | 7 (43.8%) | 0.69 | [0.22, 2.18] | .533 |  |
| Mother's education: Primary/Secondary | 20 (54.1%) | 8 (50.0%) | 0.93 | [0.30, 2.87] | .895 |  |
| Mother's education: Tertiary (reference) | 4 (10.8%) | 1 (6.3%) | — | — | — |  |
| Father's education: Any formal | 12 (32.4%) | 4 (25.0%) | 1.45 | [0.38, 5.61] | .588 |  |
| Mother is primary caregiver | 25 (67.6%) | 12 (75.0%) | 0.70 | [0.19, 2.77] | .607 |  |
| Household head: Father | 28 (75.7%) | 11 (68.8%) | 1.42 | [0.40, 5.33] | .596 |  |
| Household head: Mother | 3 (8.1%) | 2 (12.5%) | 0.62 | [0.09, 4.15] | .626 |  |
| **Exposure Variables** |  |  |  |  |  |  |
| Number of adulterated medicines (continuous) | 1.8 ± 1.0 | 1.2 ± 0.4 | 2.18 | [0.90, 5.83] | .085 | ✓ |
| Specific medicines (all listed in Table 2) |  |  |  |  |  |  |
| Promethazine | 20 (54.1%) | 4 (25.0%) | 3.51 | [1.08, 11.84] | .032 | ✓ |
| Other brand Promethazine | 5 (13.5%) | 2 (12.5%) | 1.09 | [0.18, 6.71] | .925 |  |
| Any Promethazine (combined) | 22 (59.5%) | 5 (31.3%) | 3.23 | [1.01, 10.84] | .045 | (✓) |
| **Concomitant Medications** |  |  |  |  |  |  |
| Any concomitant drug | 24 (64.9%) | 7 (43.8%) | 2.38 | [0.74, 7.82] | .143 | ✓ |
| Multivitamin | 10 (27.0%) | 9 (56.3%) | 0.29 | [0.09, 0.89] | .024 | ✓ |
| Acetaminophen | 24 (64.9%) | 6 (37.5%) | 3.06 | [0.96, 10.16] | .059 | ✓ |
| Ibuprofen | 5 (13.5%) | 1 (6.3%) | 2.33 | [0.25, 24.15] | .458 |  |
| Diclofenac | 2 (5.4%) | 0 (0.0%) | - |  | .365ᵇ |  |
| Aspirin | 0 (0.0%) | 0 (0.0%) | - | - | - |  |
| Any anti-inflammatory | 28 (75.7%) | 9 (56.3%) | 2.44 | [0.71, 8.88] | .160 | ✓ |
| Any antibiotic | 8 (21.6%) | 3 (18.8%) | 1.20 | [0.27, 5.47] | .814 |  |
| Amoxicillin | 7 (18.9%) | 2 (12.5%) | 1.63 | [0.29, 9.83] | .584 |  |
| Ampicillin | 2 (5.4%) | 0 (0.0%) | - | - | .365ᵇ |  |
| Gentamicin | 1 (2.7%) | 0 (0.0%) | - | - | .507ᵇ |  |
| Ceftriaxone | 0 (0.0%) | 1 (6.3%) | - | - | .302ᵇ |  |
| Cotrimoxazole | 1 (2.7%) | 0 (0.0%) | - | - | .507ᵇ |  |
| Ciprofloxacin | 0 (0.0%) | 0 (0.0%) | - | - | - |  |
| Other antibiotics | 1 (2.7%) | 1 (6.3%) | 0.42 | [0.02, 7.18] | .550 |  |
| Traditional oral medicine | 1 (2.7%) | 2 (12.5%) | 0.19 | [0.02, 2.15] | .156 | ✓ |
| Other cough syrup | 13 (35.1%) | 5 (31.3%) | 1.19 | [0.35, 4.14] | .779 |  |
| Herbal remedies | 3 (8.1%) | 2 (12.5%) | 0.62 | [0.09, 4.15] | .626 |  |
| Total unique medications (continuous) | 4.2 ± 2.1 | 2.8 ± 1.3 | 1.36 | [1.07, 1.74] | .012 | ✓ |
| Total medications ≥3 | 29 (78.4%) | 10 (62.5%) | 2.18 | [0.63, 7.96] | .220 |  |
| Total medications ≥6 | 11 (29.7%) | 1 (6.3%) | 6.36 | [0.75, 108.51] | .067 | ✓ |
| **Anthropometric/Nutritional** |  |  |  |  |  |  |
| Weight (kg, continuous)ᶜ | 9.8 ± 2.3 | 12.0 ± 3.1 | 0.78 | [0.54, 1.09] | .156 | ✓ |
| Height (cm, continuous)ᶜ | 62.4 ± 12.8 | 75.5 ± 15.2 | 0.96 | [0.91, 1.01] | .098 | ✓ |
| MUAC (cm, continuous)ᶜ | 13.8 ± 1.2 | 14.8 ± 1.3 | 0.58 | [0.27, 1.16] | .142 | ✓ |
| Weight-for-age <-2SD | 3 (8.1%) | 0 (0.0%) | - | - | .285ᵇ |  |
| Height-for-age <-2SD | 2 (5.4%) | 0 (0.0%) | - | - | .365ᵇ |  |
| MUAC <12.5 cm | 1 (2.7%) | 0 (0.0%) | - | - | .507ᵇ |  |
| Malnutrition (any indicator) | 2 (5.4%) | 1 (6.3%) | 0.86 | [0.07, 10.77] | .906 |  |
| **Medical History** |  |  |  |  |  |  |
| Prematurity | 4 (10.8%) | 1 (6.3%) | 1.83 | [0.18, 20.85] | .605 |  |
| Previous AKI | 0 (0.0%) | 0 (0.0%) | - | - | - |  |
| Previous hospitalization | 1 (2.7%) | 1 (6.3%) | 0.42 | [0.02, 7.18] | .550 |  |
| Previous surgery | 3 (8.1%) | 1 (6.3%) | 1.32 | [0.13, 14.32] | .822 |  |
| Chronic illness | 0 (0.0%) | 0 (0.0%) | - | - | - |  |
| **Environmental Exposures** |  |  |  |  |  |  |
| Flooding in residential area | 3 (8.1%) | 1 (6.3%) | 1.32 | [0.13, 14.32] | .822 |  |
| Pesticide exposure | 1 (2.7%) | 0 (0.0%) | - | - | .507ᵇ |  |
| Herbicide exposure | 0 (0.0%) | 0 (0.0%) | - | - | - |  |
| Industrial chemical exposure | 0 (0.0%) | 0 (0.0%) | - | - | - |  |
| Natural toxins | 0 (0.0%) | 0 (0.0%) | - | - | - |  |
| Animal bite/sting | 0 (0.0%) | 0 (0.0%) | - | - | - |  |

*Note.* OR = odds ratio; CI = confidence interval; WR1 = Western Region 1; WR2 = Western Region 2; URR = Upper River Region; MUAC = mid-upper arm circumference; SD = standard deviation; AKI = acute kidney injury.

ᵃVariables with *p* ≤ .20 selected as candidates for multivariable modeling. Checkmark (✓) indicates selection; (✓) indicates considered but not included due to collinearity with selected variable.

ᵇ*p*-value from Fisher's exact test (cell count = 0).

ᶜAnthropometric data available for limited sample (Exposed-Susceptible: *n* = 11; Exposed-Resistant: *n* = 6).

**Table J: Model Comparison and Goodness-of-Fit Statistics for Multivariable Logistic Regression Models**

| **Model Specification** | **Variables Included** | ***n*** | **-2LL** | **AIC** | **BIC** | **Pseudo-*R*²** | **Hosmer-Lemeshow** | **AUC (95% CI)** | **Brier Score** |
| --- | --- | --- | --- | --- | --- | --- | --- | --- | --- |
| **Null Model** |  |  |  |  |  |  |  |  |  |
| Intercept only | - | 53 | 67.42 | 69.42 | 71.28 | - | - | 0.50 | 0.232 |
| **Single-Predictor Models** |  |  |  |  |  |  |  |  |  |
| Age only | Age (per 12 mo) | 53 | 58.28 | 62.28 | 65.99 | .136 | 8.24 (.410) | 0.71 (0.57–0.85) | 0.210 |
| Multivitamin only | Multivitamin use | 53 | 62.15 | 66.15 | 69.86 | .078 | 3.12 (.873) | 0.64 (0.49–0.79) | 0.220 |
| Medicines only | ≥2 medicines | 53 | 63.82 | 67.82 | 71.53 | .054 | 2.85 (.827) | 0.61 (0.46–0.76) | 0.224 |
| **Two-Variable Models** |  |  |  |  |  |  |  |  |  |
| Age + Multivitamin | Age, Multivitamin | 53 | 52.36 | 58.36 | 63.93 | .223 | 6.82 (.556) | 0.78 (0.65–0.91) | 0.192 |
| Age + Medicines | Age, ≥2 medicines | 53 | 53.12 | 59.12 | 64.69 | .212 | 7.15 (.520) | 0.76 (0.63–0.89) | 0.195 |
| Multivitamin + Medicines | Multivitamin, ≥2 medicines | 53 | 57.28 | 63.28 | 68.85 | .151 | 4.28 (.748) | 0.72 (0.58–0.86) | 0.204 |
| **Full Models** |  |  |  |  |  |  |  |  |  |
| Model 1 (Primary) | Age, Sex, ≥2 medicines, Multivitamin | 53 | 45.82 | 55.82 | 64.82 | .321 | 6.12 (.634) | 0.82 (0.70–0.95) | 0.178 |
| Model 1a (Saturated) | Model 1 + Region + Income | 53 | 42.18 | 58.18 | 72.75 | .373 | 5.84 (.665) | 0.84 (0.72–0.96) | 0.172 |
| Model 2 (Medicine-specific) | Age, Sex, Promethazine, Multivitamin | 53 | 46.52 | 56.52 | 65.52 | .312 | 5.89 (.659) | 0.81 (0.68–0.94) | 0.180 |
| Model 3 (Total burden) | Age, Sex, Total meds (cont), Multivitamin | 53 | 47.85 | 57.85 | 66.85 | .290 | 7.28 (.508) | 0.79 (0.66–0.92) | 0.184 |
| Model 4 (Interaction) | Model 1 + Age×Medicines interaction | 53 | 44.68 | 56.68 | 67.54 | .338 | 6.45 (.597) | 0.83 (0.71–0.96) | 0.175 |
| **Model Selection Criteria** |  |  |  |  |  |  |  |  |  |
| Best by AIC | Model 1 (Primary) | - | - | **55.82** | - | - | - | - | - |
| Best by BIC | Model 1 (Primary) | - | - | - | **64.82** | - | - | - | - |
| Best by AUC | Model 4 (Interaction) | - | - | - | - | - | - | **0.83** | - |
| Best by Brier Score | Model 1a (Saturated) | - | - | - | - | - | - | - | **0.172** |
| **Recommended Model** | **Model 1** (Primary) | - | Parsimony, good fit, interpretable |  |  |  |  |  |  |

*Note.* -2LL = -2 log-likelihood; AIC = Akaike information criterion; BIC = Bayesian information criterion; AUC = area under the receiver operating characteristic curve; CI = confidence interval; mo = months; cont = continuous. Hosmer-Lemeshow statistic presented as χ² (*p*-value); good fit indicated by *p* > .05. Pseudo-*R*² is Nagelkerke's *R*². Brier score measures calibration (lower is better; range 0–1). Model 1 (primary model) selected based on balance of parsimony (lowest AIC/BIC), good discrimination (AUC = 0.82), adequate calibration (Hosmer-Lemeshow *p* = .634), and interpretability.

**Model selection rationale:** Model 1 was selected as the primary model for reporting based on multiple criteria: (1) lowest AIC and BIC among models without interaction terms, indicating an optimal balance of fit and parsimony; (2) excellent discrimination (AUC = 0.82); (3) adequate calibration (Hosmer-Lemeshow *p* = .634); and (4) interpretability with clinically meaningful variables. Model 1a (saturated) improved the fit marginally but added complexity without a substantial gain in AUC (0.84 vs. 0.82). Model 4 (interaction) had the highest AUC, but the interaction term was not significant (*p* = .284, see Table G), and the model was more complex. Model 1 provides the best combination of statistical performance and clinical utility for identifying protective factors among exposed children.
